# Fabrication of Electropolymerized Binder-Free Flexible Electrode of PANI-Fe-Doped *Borassus flabellifer* *-*Biomass-Derived (0D)-Carbon Quantum Dots for High-Performance Asymmetric Supercapacitors

**DOI:** 10.1021/acsomega.5c06469

**Published:** 2025-12-02

**Authors:** Nithesh Kumar Krishnan, Esakkimuthu Shanmugasundaram, Harini Bhagyaraj, Kannan Vellaisamy, Amos Ravi, As’ad Ibrahim, Na’il Saleh, Stalin Thambusamy

**Affiliations:** † Department of Industrial Chemistry, 29942 Alagappa University, Karaikudi 630 003, Tamil Nadu India; ‡ Department of Chemistry, Thiagarajar College of Engineering, Madurai 625015, Tamil Nadu, India; § Department of Materials Science, 242279 Central University of Tamil Nadu, Thiruvarur 610 005, Tamil Nadu, India; ∥ Department of Chemistry, College of Science, 11239 United Arab Emirates University, P.O. Box 15551 Al Ain, United Arab Emirates

## Abstract

The rapid evolution of energy storage technologies demands electrode materials that deliver a high energy density, flexibility, and long-term stability. In this work, a binder-free, flexible supercapacitor electrode was fabricated via electro-polymerization of polyaniline (PANI) doped with Fe ions and carbon quantum dots (CQD). The novelty of this study lies in the first-time use of biomass-derived CQDs from *Borassus flabellifer*, which exhibit a strong natural affinity toward Fe^3+^ ions, enabling uniform incorporation into the PANI matrix during electro-polymerization. This unique design integrates three complementary charge-storage mechanisms by high conductivity from the PANI backbone, reversible Fe^3+^ redox activity, and EDLC-type capacitance from the CQD, along with their role as conductive spacers to stabilize Fe^3+^ redox centers and prevent polymer aggregation. Owing to this synergy, the PANI-Fe-CQD polymerized on the CC electrode achieved a high areal capacitance of 1212.5 mF/cm^2^ at 1 mA/cm^2^ with 86.9% retention after 5000 cycles, while an asymmetric device (AC//PVA-H_2_SO_4_//PANI-Fe-CQD) delivered an energy density of 53.25 μWh/cm^2^ at a power density of 0.9 mW/cm^2^ and retained 91.2% capacitance after 5000 cycles. This work possesses a novel strategy of integrating biomass-derived *B. flabellifer* CQDs, not only replacing the need for binders and conductive agents but also enhancing the electrochemical performance of flexible electrodes, offering new directions for sustainable, high-performance energy storage devices.

## Graphical abstract

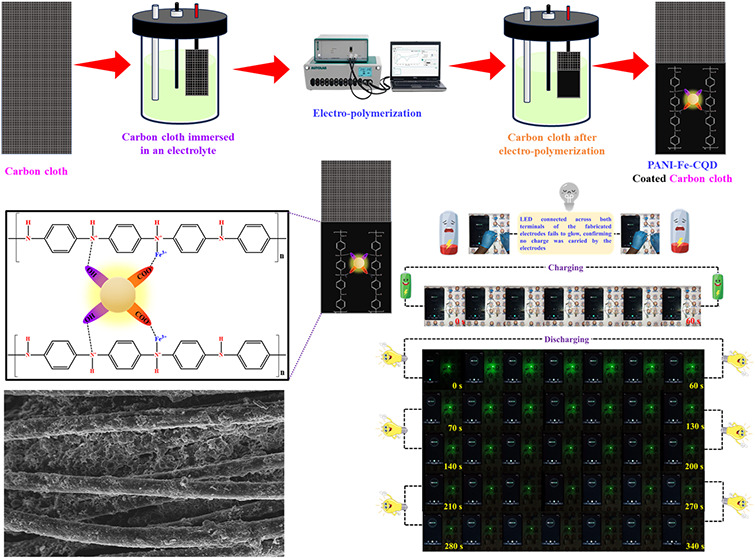

## 1 Introduction

The energy generation and energy conversion systems are increasing worldwide day by day due to the increase in global energy demand.^1^ Even though the energy systems are generated, the energy storage devices, like batteries,^2,3^ can store and deliver high energy density.^4^ To overcome this, supercapacitors are developed and can deliver a high amount of power (high-power density),^5^ a longer life cycle,^6^ and possess low resistance. Usually, based on the types of materials used, the supercapacitors are split up into electrical double-layer capacitors (EDLCs)^7^ and pseudocapacitors.^8^ So far, in recent years, versatile materials have been prepared to enhance the capacitance behavior of the supercapacitors. Maniyazagan et.al. have developed an S/CoMnP@C, a metal organic framework-doped sulfur for asymmetric supercapacitor application. The CoMnP@C was coated on the Nickel foam substrate and possesses 1600 F g-1 at 1 A g-1 and is stable over 500 cycles with the capacitance retention over 96%. The prepared asymmetric device delivers 850.53 W kg^–1^ power density and 37.32 W h kg^–1^ energy density, respectively.^9^ Munde et.al. have developed Cr-doped NiO/Fe_2_O_3_ nanomaterials that deliver a maximum specific capacitance of 1595.6 Fg^1–^ at 10 mV/s, and with 82% capacitance retention after 2000 cycles, this enhanced performance corresponds to the doping of Cr to the NiO/Fe_2_O_3_ matrix.^10^ Tourchi Moghadam et al. have prepared a ZnWO_4_–CNT coated on a nickel substrate and tested with 3 M KOH electrolyte. The ZnWO_4_–CNT possesses 4552 F g^–1^ at 1 A g^–1^, the cyclic stability withstands 92% over 3000 cycles in 100 mV/s, and the prepared asymmetric supercapacitor device analysis performed with ZnWO_4_–CNT as positive and RGO/NF as negative electrodes delivers about a high capacitance of 320 F g^–1^ with cycling stability of 78% after 3000 consecutive cycles.^11^ In conventional electrode fabrication, the electroactive materials are typically coated on a current collector such as Ni-foam, Cu-foam, or Ni-mesh. This process usually requires the addition of a conductive agent (e.g., acetylene black) and a polymer binder (e.g., PVDF) to ensure proper adhesion and conductivity.

Although certain energy storage materials exhibit high specific capacitance, they often fall short when it comes to flexibility, limiting their applicability in next-generation flexible and wearable electronics. In recent years, there has been a significant surge in research focused on developing flexible supercapacitor materials that can combine a high energy storage capacity with mechanical flexibility. This growing interest stems from the increasing demand for lightweight, foldable, and stretchable energy storage devices that can seamlessly integrate with portable and wearable technologies, and a novel electro-polymerization technique was used to prepare the flexible and binder-free supercapacitor devices. Shanmugasundaram et.al. have fabricated a carbon quantum dot-doped PANI for organic solar cell application.^12^ Polypyrrole (PPY),^13,14^ polythiophene (PTH),^15^ and polyaniline (PANI)^16,17^ are the commonly known conducting polymers that are widely used in supercapacitor applications due to their superior electrical conductivity properties, cost-intensive properties, and iron and CQD^18^ are employed as dopants along with PANI to enhance the electrical properties and the charge–discharge mechanisms.

Carbon quantum dots are zero-dimensional materials (0D) with a size less than 10 nm and have gained attention due to their fluorescent and electroactivity properties. Xu et.al. have synthesized the carbon quantum dot while purifying single-walled carbon nanotubes.^19^ Chen et.al*.*, have developed a PANI doped CQD Via a spraying method using FTO/glass plate as a substrate for enhancing the electrochromic supercapacitor and stable over 5000 cycles.^20^ Shanmugasundaram et.al*.*, constructed a binder-free PANI-CQD-Cu in carbon cloth for enhancing the supercapacitor application that delivers the areal capacitance of 1070 F g^–1^ at 1 A g^–1^, and the prepared asymmetric supercapacitor AC/PVA-H_2_SO_4_//PANI-CQD-Cu delivers energy and power densities of 23.10 μW h cm^–2^ and 0.978 mW cm^–2^, respectively, and possesses a capacitance retention over 92% over 3000 cycles.^21^ Can Zhou et al. have fabricated the CQD/PPy on cotton cloth as a binder-free flexible electrode supercapacitor, delivering a specific capacitance of 537.9 F/g at 0.5 A/g, and the asymmetric supercapacitor device has expressed a high-power density of 125.0 W/kg and energy density of 18.7 Wh/kg, and the symmetric devices was stable over 10,000 cycles with capacitance retention over 77.9%.^22^ Compared to the chemically synthesized CQDs, the biomass-derived CQD exhibits enhanced electrochemical performance. The major reason for doping CQD with Fe via electro-polymerization is mainly due to CQD having a natural affinity to bind with Fe metal ions,^23,24^ and during the fabrication of the electrode by electro-polymerization, the CQD can easily interact with the Fe ions. The CQDs, synthesized from *Borassus flabellifer* biomass for the first time, possess abundant oxygen groups that exhibit a strong natural affinity toward Fe^3+^ ions. The carbon-based CQDs become uniformly embedded within the PANI matrix, where they stabilize Fe redox centers, act as conductive spacers to prevent polymer chain aggregation, and contribute additional double-layer capacitance. Furthermore, electron transport occurs through the PANI backbone, pseudo capacitance arises from the reversible Fe^3+^ redox process, and double-layer charge storage is provided by the CQDs. The CQDs prepared from biomass-derived CQDs possess advantages over chemical synthesis methods. The chemical routes require high-cost reagents and precursors and some complex steps in the synthesis, whereas the biomass-derived sources are renewable, inexpensive, and eco-friendly. Moreover, the inherent oxygen- and nitrogen-containing functional groups present in natural precursors facilitate excellent surface functionalization and superior dispersibility in aqueous media without additional chemical treatment. The size-controlled morphology obtained from biomass precursors possesses better homogeneity and crystallinity, as observed in the TEM images. The synergistic interactions among these three components enhance the redox process, charge storage behavior, and cycling stability, thereby improving the overall electrochemical performance. So with this theoretical evidence, this work aims to synthesize CQD from *B. flabellifer* and to prepare a PANI-Fe-CQD binder-free flexible electrode, and characterize it with structural and electrochemical studies.

## 2 Experimental Section

### 2.1 Materials Required

The materials required are stated in the Supporting Information.

### 2.2 Experimental Procedure

#### 2.2.1 Synthesis of Carbon Quantum Dots (CQDs)

5 g portion of powder of spadix from *B. flabellifer* (flower of male palm tree) was dried in the oven for 12 h. The powder was dissolved in 20 mL of ethanol and 20 mL of water (1:1). The solution was placed in a Teflon-stainless steel autoclave and heated to 180 °C for 6 h. A brown solution was obtained and washed with dichloromethane. The aqueous solution was subjected to dialysis for 2 days and used for further studies.^25^ The synthesis method is schematically illustrated in Figure S1.

#### 2.2.2 Preparation of PANI-Fe-CQD

To fabricate the PANI-Fe-CQD electrode, the electro-polymerization process was carried out using a 1 × 1 cm^2^ piece of carbon cloth as the working electrode, an Ag/AgCl electrode as the reference, and a platinum wire as the counter electrode. A solution containing 0.2 M aniline, 0.2 M ferrous sulfate (FeSO_4_), 2 mL of CQD, and 0.5 M sulfuric acid was prepared in 50 mL of deionized water to serve as the electrolyte. The polymerization was conducted by the potential window from −0.2 V to +0.8 V at a scan rate of 50 mV/s for 10 cycles. Followed by electro-polymerization, the modified carbon cloth was dried at 50 °C for 3 h, as shown in Scheme 1. The same procedure was employed to fabricate the PANI and PANI-Fe electrodes, excluding the respective components, as required. The CV polymerized curves for the PANI, PANI-Fe, and PANI-Fe-CQD are illustrated in Figure S1.

**Scheme 1:**
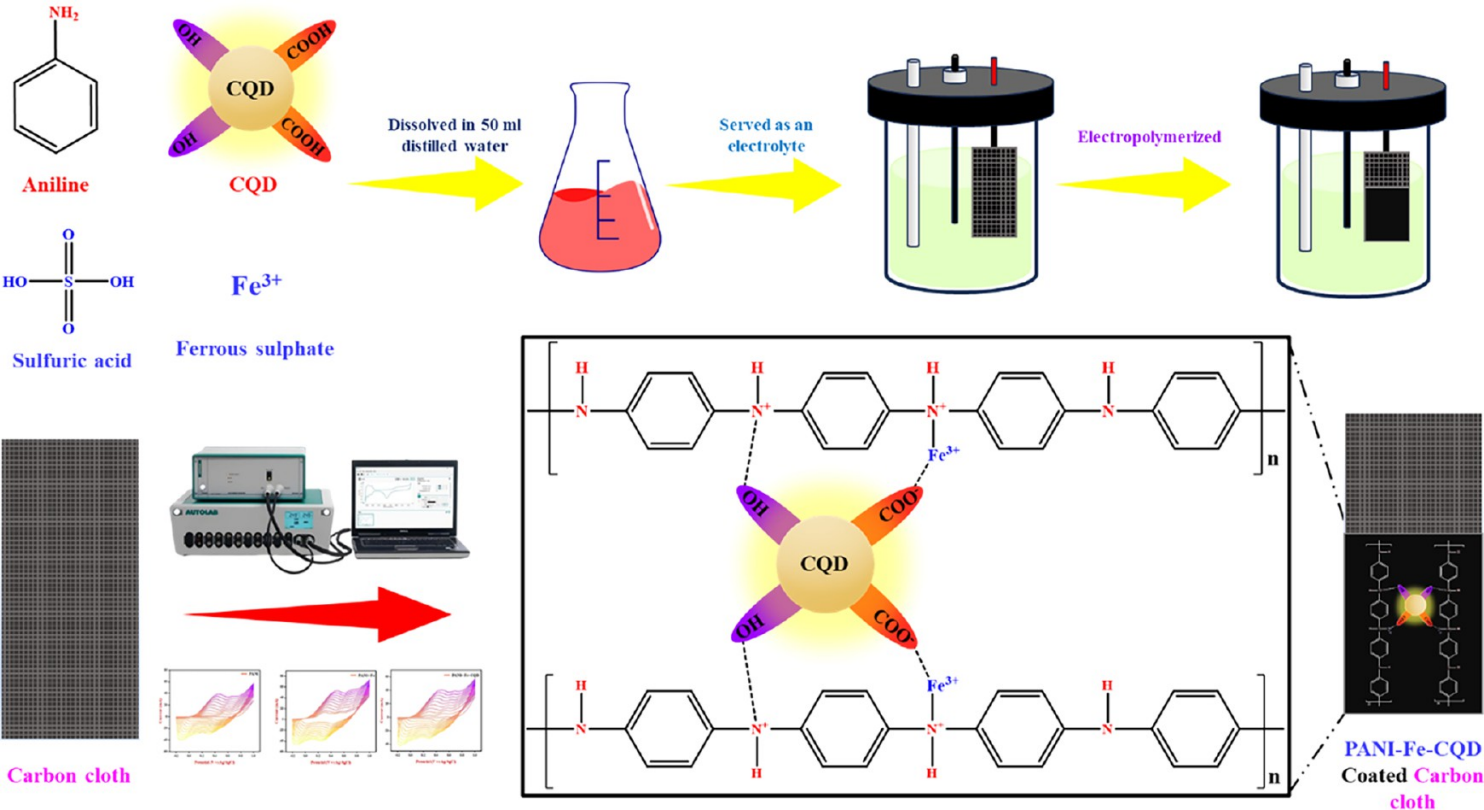
Fabrication of PANI, PANI-Fe, PANI-Fe-CQD Electrodes Using Electro-Polymerization

#### 2.2.3 Preparation of Flexible Asymmetry Supercapacitor

To fabricate the flexible asymmetric supercapacitor, the PANI-Fe-CQD-coated carbon cloth served as the cathode, while an activated carbon-coated carbon cloth functioned as the anode with a polymer gel electrolyte acting as the separator. For preparing the solid quasi-electrolyte, in 20 mL of hot water, 1 g of poly­(vinyl alcohol) (PVA) was dissolved. In which 0.5 M sulfuric acid solution was added. The mixture was continuously stirred and heated at 80 °C until a clear gel formed. Before device assembly, the gel was gently coated on both electrodes and then allowed to dry. Afterward, the cathode and anode were aligned with the PVA//H_2_SO_4_ gel sandwiched between them, and the entire setup was sealed using paraffin film. The assembled device was then evaluated by electrochemical measurements.

### 2.3 Characterization Techniques

The characterization techniques are discussed in the Supporting Information.

## 3 Results and Discussions

### 3.1 Structural Studies of CQDs

#### 3.1.1 Optical and FT-IR Studies

The UV–vis absorption spectrum of the synthesized CQD, as shown in Figure 1(a), exhibits distinct absorption peaks at 203 and 310 nm, attributed to the π-π* transition and n-π* transition corresponding to the presence of (C–C), (C=C) carbonyl (C=O) functional groups on the CQD surface. This peak indicates the presence of oxygen-containing functionalities, confirming surface oxidation during the synthesis process. These surface groups are crucial as they influence the electronic structure and optical response of the CQDs. The fluorescence excitation-dependent emission spectra were recorded by varying the excitation wavelength from 250 to 360 nm, as presented in Figure 1(b). A blue shift in the emission maxima was observed, with the highest intensity at 330 nm, followed by a gradual decrease in intensity at higher excitation wavelengths. This excitation-dependent fluorescence is characteristic of CQDs and is attributed to multiple emission centers arising from surface defect states, particle size distribution, and π-π* transitions of the sp^2^-hybridized carbon core.^26^ The presence of these diverse emissive sites and energy levels suggests that CQDs exhibit strong and tunable fluorescence, making them ideal for sensing applications.

**Figure 1:**
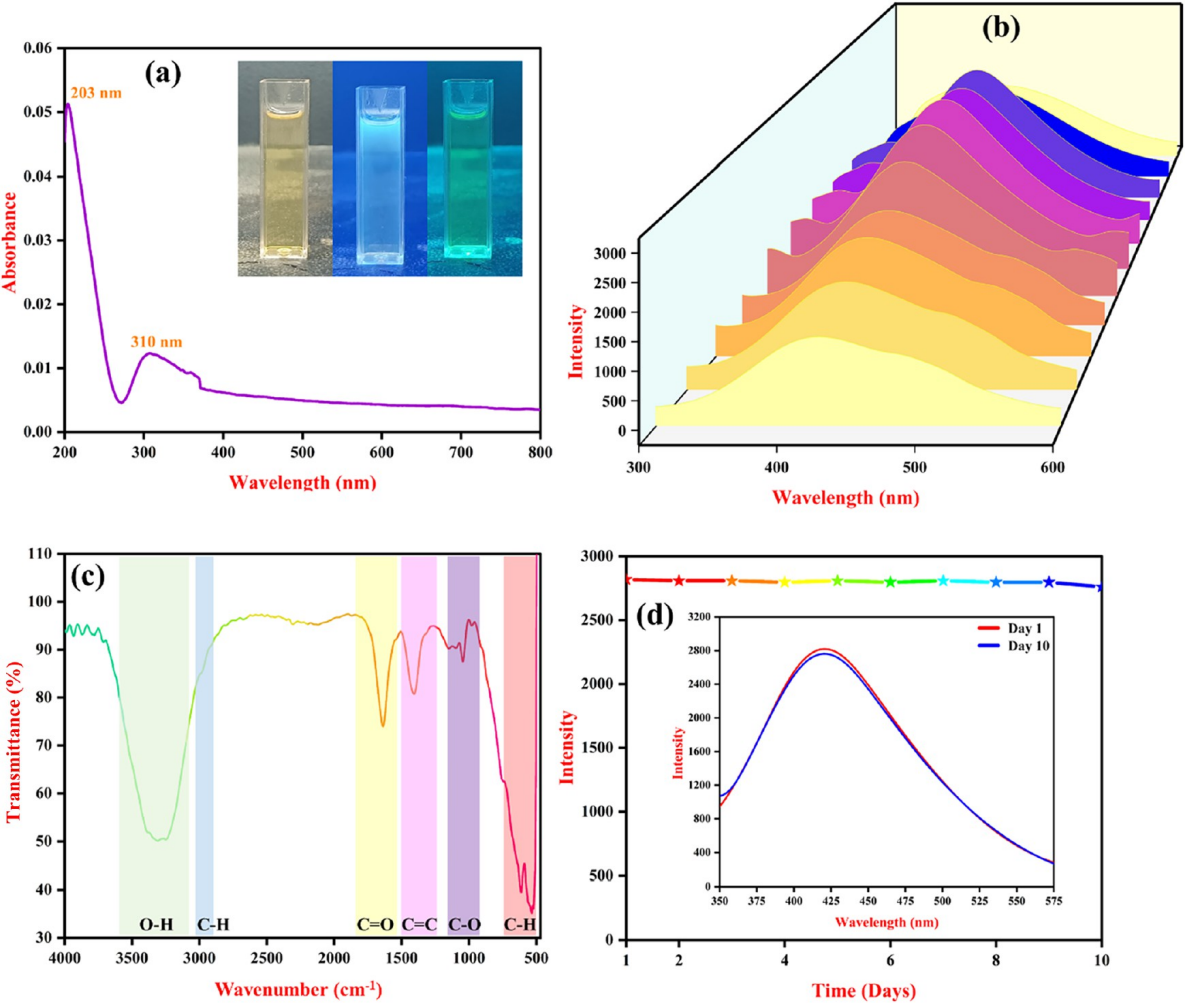
Optical studies of CQD in (a) UV–vis spectroscopy (inset: visual image of CQD in visible and UV lights), (b) fluorescence spectra with different excitation from 250 to 360 nm, (c) FT-IR studies of CQD, and (d) fluorescence stability of CQD (inset: fluorescence spectra of day 1 and 10).

The presence of functional groups on the CQD surface was confirmed by using Fourier-transform infrared (FT-IR) spectroscopy (Figure 1(c)). A broad and intense peak around 3323 cm^–1^ corresponds to O–H stretching vibrations,^27,28^ indicating the presence of hydroxyl groups.^29^ The peak at 1639 cm^–1^ is assigned to the C=O stretching vibration,^30^ while the peak at 1048 cm^–1^ corresponds to the C–O stretching,^28^ confirming the existence of ether or alcohol groups. Additionally, the aromatic C–H bending vibrations observed at 675 cm^–1^ further suggest the presence of an aromatic carbon structure.^31^ In Figure 1(d), the fluorescence emission of the CQDs remained constant from day 1 to day 10, indicating excellent photostability under ambient conditions. This long-term stability can be attributed to the presence of various surface functional groups, such as hydroxyl (−OH), carbonyl (C=O), and alkyl/aryl (C–C) bonds. These functional groups play a critical role in stabilizing the electronic structure of CQD by passivating surface trap states and preventing photoinduced degradation. The −OH and C=O groups enhance solubility and create hydrogen bonding interactions with the surrounding environment, which protect the CQD core from oxidative or photochemical damage. Meanwhile, the robust C–C framework contributes to the structural integrity and resilience of the CQD. Together, these functionalities ensure minimal fluctuation in the fluorescence intensity, confirming the chemical and photophysical stability of the CQD over time.

#### 3.1.2 Transmission Electron Microscope (TEM) Analysis

The size distribution and surface morphology of the CQDs were examined using TEM, as illustrated in Figure 2. Figure 2(a–e) displays TEM images of the CQDs at different magnifications, 200, 100, 50, 20, and 10 nm, respectively. These images reveal that the CQDs are well-dispersed in solution and exhibit a spherical morphology.^32^ The particle sizes range from 2 to 10 nm, and the particle size distribution, determined from the image analysis, is approximately 5.367 nm, as shown in Figure S2.

**Figure 2:**
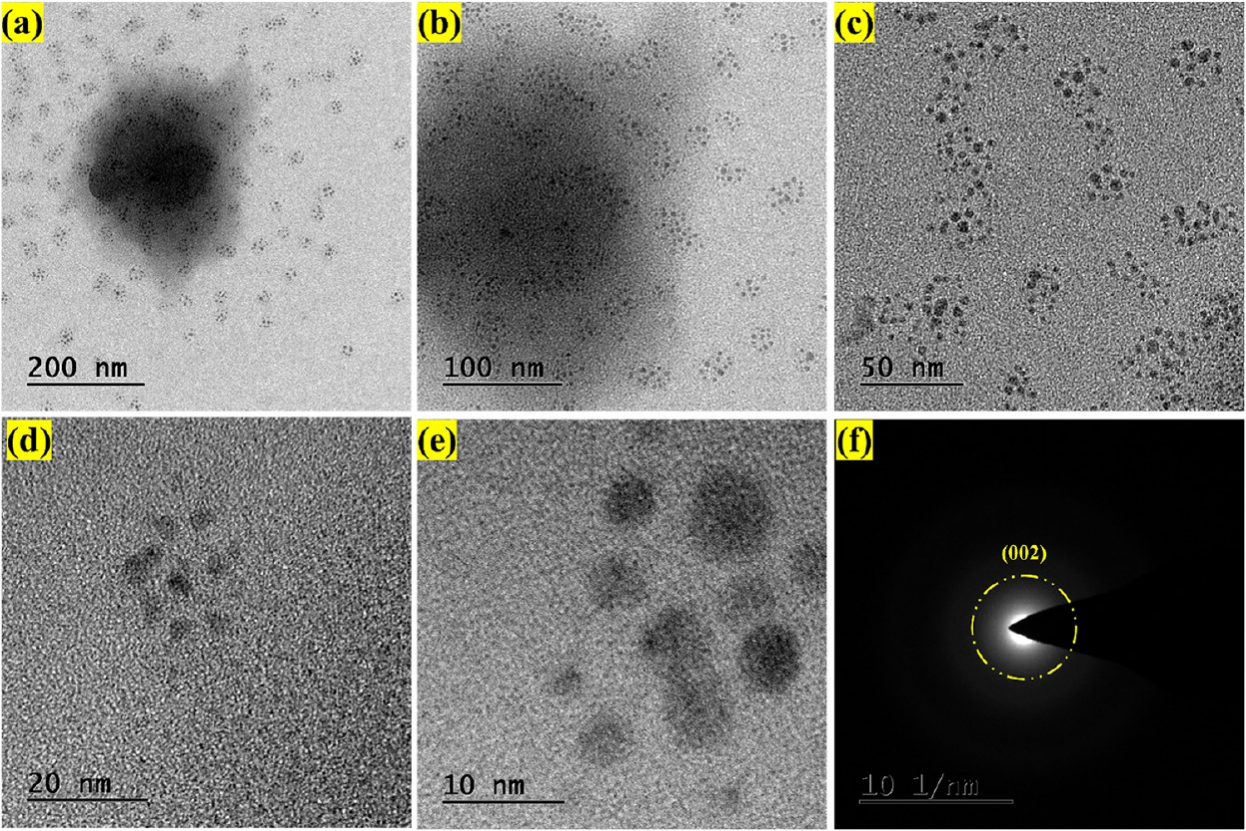
TEM morphology of the CQD at (a) 200 nm, (b) 100, (c) 50, (d) 20, (e) 10 nm, and (f) SAED pattern of CQD.

Figure 2(f) presents the SAED pattern of the CQDs. The observed diffraction ring corresponds to the (002) plane of graphitic carbon, indicating the partial crystallinity (amorphous) nature of the CQDs. The broad and diffuse nature of the diffraction rings also suggests an amorphous carbon matrix. This combination of graphitic domains and amorphous structures is characteristic of CQDs, and this confirms the presence of carbon as the primary constituent within the CQD framework.

### 3.2 Structural Studies of Fabricated Flexible Electrodes

#### 3.2.1 X-ray Diffraction Analysis (XRD) and Raman Analysis

The crystalline and structural features of the fabricated PANI, PANI-Fe, and PANI-Fe-CQD flexible electrodes were analyzed by using X-ray diffraction XRD. Figure 3(a) displays that the PANI displayed characteristic diffraction peaks at approximately 24.88° and 42.36°, corresponding to the (002) and (101) planes, and indicating the semicrystalline structure typically associated with the ordered stacking of the polymer chains. The broad and intense nature of these peaks also reflects the presence of a disordered amorphous region, attributed to the repeating benzenoid and quinoid units along the PANI polymeric backbone.^33^ Upon interaction with Fe, the PANI matrix formed PANI-Fe, and an additional diffraction peak appeared at 31.25°, confirming the successful coordination or doping of Fe species that interact with nitrogen-containing functional groups -NH- or = N- present in the PANI chains through coordination bonding or electrostatic interactions. In the case of the PANI-Fe-CQD composite, the diffraction pattern showed a slight shift in peak positions, indicating structural distortion due to the integration of CQD in the PANI matrix, and this shift is due to the interaction between surface functional groups of the CQDs, such as carboxyl (−COOH) and hydroxyl (−OH), and the PANI-Fe network. These interactions can slightly alter the lattice parameters and affect the degree of crystallinity of the composite.^34^

**Figure 3:**
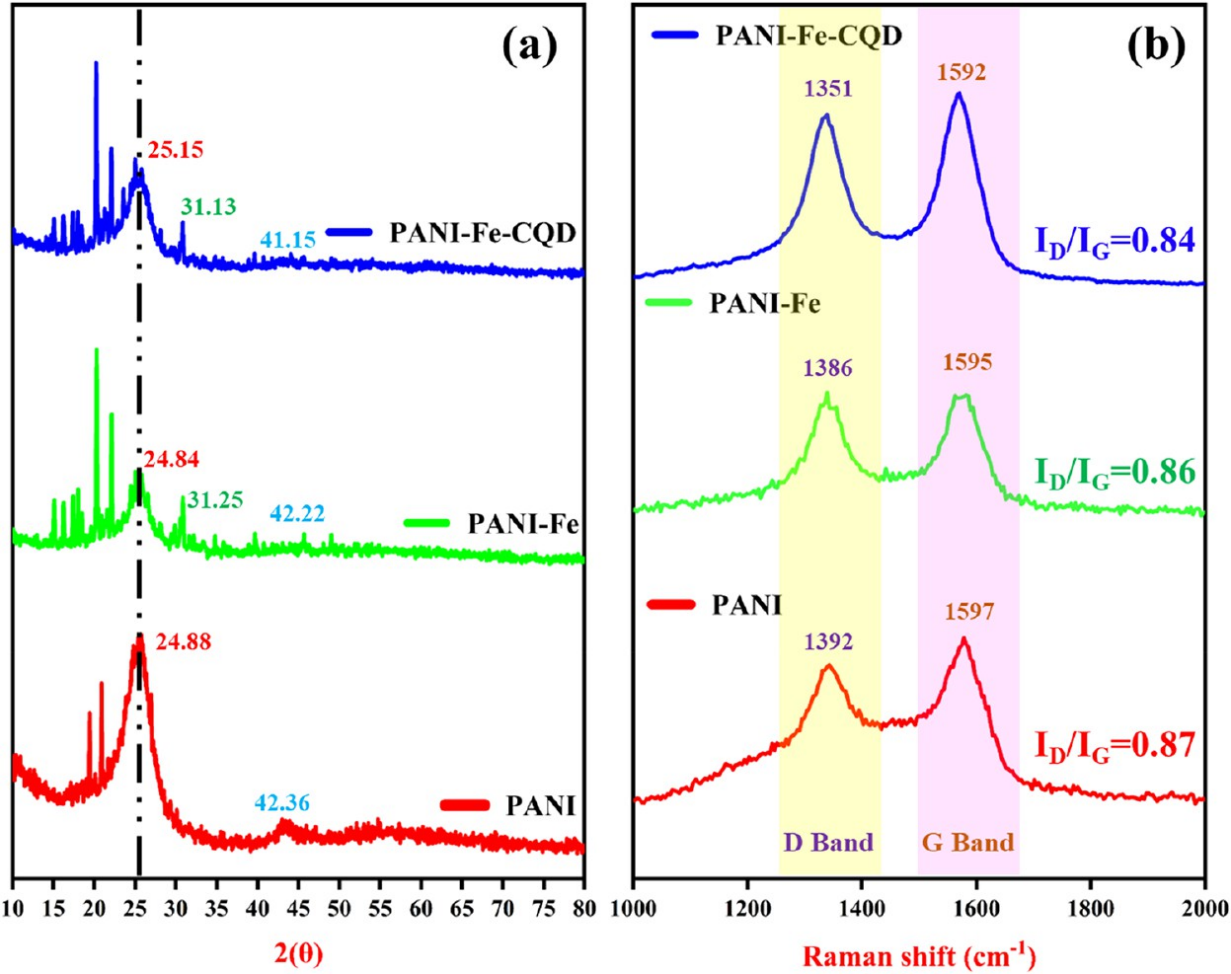
(a) XRD diffraction pattern. (b) Raman spectra of PANI, PANI-Fe, and PANI-Fe-CQD.

Figure 3(b) presents the Raman spectra of the polymerized electrodes, providing insights into the structural order and defect density of the fabricated materials. Raman spectroscopy is a widely used method to study disorder and carbon-based bonding in conjugated systems. In this context, the D band (∼1350–1390 cm^–1^) corresponds to the A_1g_ breathing mode associated with defects, disorder, or edge carbon atoms, while the G band (∼1590–1600 cm^–1^) arises from the *E*_g_ in-plane stretching of sp^2^-hybridized carbon atoms, reflecting graphitic order.^35^ The PANI exhibits the D and G bands at 1392 and 1597 cm^–1^, respectively, with a calculated *I*_D_/*I*_G_ ratio of 0.87, indicating a moderate level of structural disorder typical of conjugated polymers. Upon incorporation of Fe, the PANI-Fe electrode shows a slight red shift of both bands (D at 1386 cm^–1^ and G at 1595 cm^–1^), and a marginally reduced *I*_D_/*I*_G_ value of 0.86. This suggests minor reorganization of the polymer backbone due to Fe–N coordination interactions, where Fe ions may interact with the imine (=N-) and amine (−NH-) groups of PANI, enhancing conjugation and slightly reducing defect density. And the PANI-Fe-CQD composite exhibits further shifts, with the D and G bands appearing at 1351 and 1592 cm^–1^, and a more pronounced decrease in the *I*_D_/*I*_G_ ratio to 0.84. This decrease suggests an increase in graphitic ordering, likely due to π–π stacking interactions between the aromatic domains of CQDs and the conjugated backbone of PANI.^36^ Additionally, the functional groups in the CQD form noncovalent interactions with nitrogen functionalities in PANI and coordinate with Fe^3+^ centers. These interactions help to bridge the components, improving the structural integration and reducing edge defects, which results in a lower defect-related D band intensity.

#### 3.2.2 Scanning Electron Microscope (SEM) Analysis

The morphology of the fabricated flexible electrodes was systematically analyzed using SEM. SEM images illustrated in Figure 4(a–c) depict the morphology of PANI coated on carbon cloth (CC) at varying magnifications (20 μm, 10 μm, and 5 μm). These images reveal that PANI forms a continuous and well-adhered layer over the fibrous structure of the carbon cloth, indicating successful deposition and uniform coverage. Moving on to the PANI-Fe composite, Figure 4(d–f) displays the SEM images that exhibit the integration of iron (Fe) species within the PANI matrix. The micrographs suggest that Fe is uniformly distributed throughout the polymer, with no significant aggregation. This homogeneous distribution implies effective coordination between Fe^3+^ ions and the nitrogen sites in the PANI backbone, likely through coordination bonding or electrostatic interactions. Such binding not only supports structural integrity but may also enhance the electrochemical performance.^37^ Further, Figure 4(g–i) illustrates the morphology of the PANI-Fe-CQDs. The images clearly show the uniformly distributed, spherical CQDs decorating the electrode surface. The even coverage and tight binding of CQDs suggest strong interactions, potentially via π–π stacking between the aromatic structures of PANI and the graphitic nature of the CQDs. These interactions facilitate good adhesion and efficient charge transfer at the interface, play a vital role in improving the overall conductivity and functionality of the electrode^38^ Figure S3 displays the SEM morphology before and after electrochemical performance. It reveals that there are no morphological changes observed after the electrochemical experiment, thereby indicating a stable interaction between the PANI-Fe-CQD coated on the Carbon cloth.

**Figure 4:**
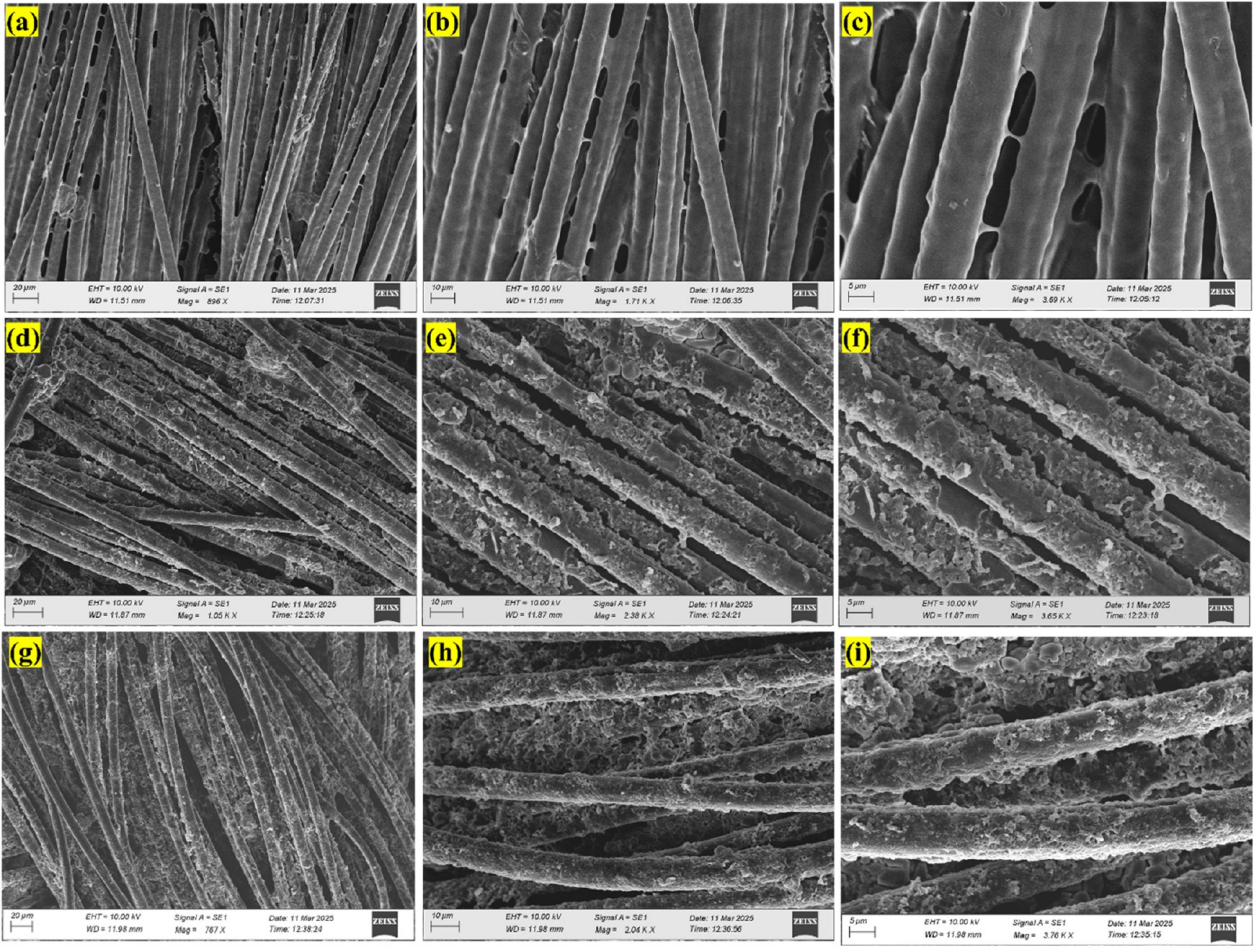
SEM morphology of (a-c) PANI, (d-f) PANI-Fe, and (g-i) PANI-Fe-CQD at different magnifications of 20 μm, 10 μm, and 5 μm, respectively.

#### 3.2.3 Energy Dispersive X-ray Analysis (EDAX) and Elemental Analysis

The elemental compositions of the PANI, PANI-Fe, and PANI-Fe-CQD electrodes were thoroughly examined through EDAX and elemental line mapping. Figure 5(a) presents the EDAX spectrum for the electropolymerized PANI electrode. The analysis shows significant contents of carbon (63.97%) and nitrogen (36.03%), consistent with the expected composition of polyaniline chains formed during the electro-polymerization of aniline monomers. In Figure 5(a­(i–iv)), elemental mapping images are presented. Figure 5(a-i) depicts the scanned electrode area, Figure 5(a-ii) shows the total elemental distribution, while Figure 5(a-iii) and Figure 5(a-iv) distinctly map the distribution of carbon (C) and nitrogen (N), respectively. The uniform C and N mapping reveals the homogeneous electro-polymerization process across the electrode surface. Figure 5(b) shows the EDAX spectrum of the PANI-Fe electrode, where additional elemental signals emerge after Fe incorporation. The spectrum indicates the presence of iron (Fe) at 15.04%, along with carbon (23.72%) and nitrogen (14.06%). This confirms that Fe species have been successfully introduced during or after the electro-polymerization process. During polymerization, Fe ions are either physically entrapped within the growing PANI matrix or weakly coordinated through interactions with nitrogen sites of PANI, without forming a rigid framework. Figure 5(b­(i-v)) depicts the elemental mapping results. Figure 5(bi-ii) illustrates the surveyed area and overall elemental distribution, while Figure 5 (biii-v) separately maps carbon, nitrogen, and iron. The relatively uniform distribution of Fe suggests effective dispersion of Fe species throughout the polymeric network. Figure 5(c) provides the EDAX spectrum of the PANI-Fe-CQD composite. Compared with PANI-Fe, the spectrum shows notable amounts of carbon (18.19%), nitrogen (6.88%), oxygen (33.44%), and iron (13.52%). The significant oxygen content and reduced carbon and nitrogen percentages strongly imply the incorporation of oxygen-rich CQDs onto the electropolymerized PANI-Fe surface. The mechanism likely involves surface adsorption or entanglement of CQDs within the porous electropolymerized matrix rather than chemical covalent bonding. The oxygenated functional groups (−COOH, −OH) on CQDs can form hydrogen bonds or electrostatic interactions with PANI and Fe sites, facilitating a strong physical association. The elemental mappings shown in Figure 5(c­(i-vi)) include the scanned area, Figure 5(c­(i)), full elemental mapping, Figure 5(c­(ii)), the separate mappings of carbon, Figure 5(c­(iii)), nitrogen, Figure 5(c­(iv)), oxygen, Figure 5(c­(v)), and iron, Figure 5(c­(vi)), respectively.

**Figure 5:**
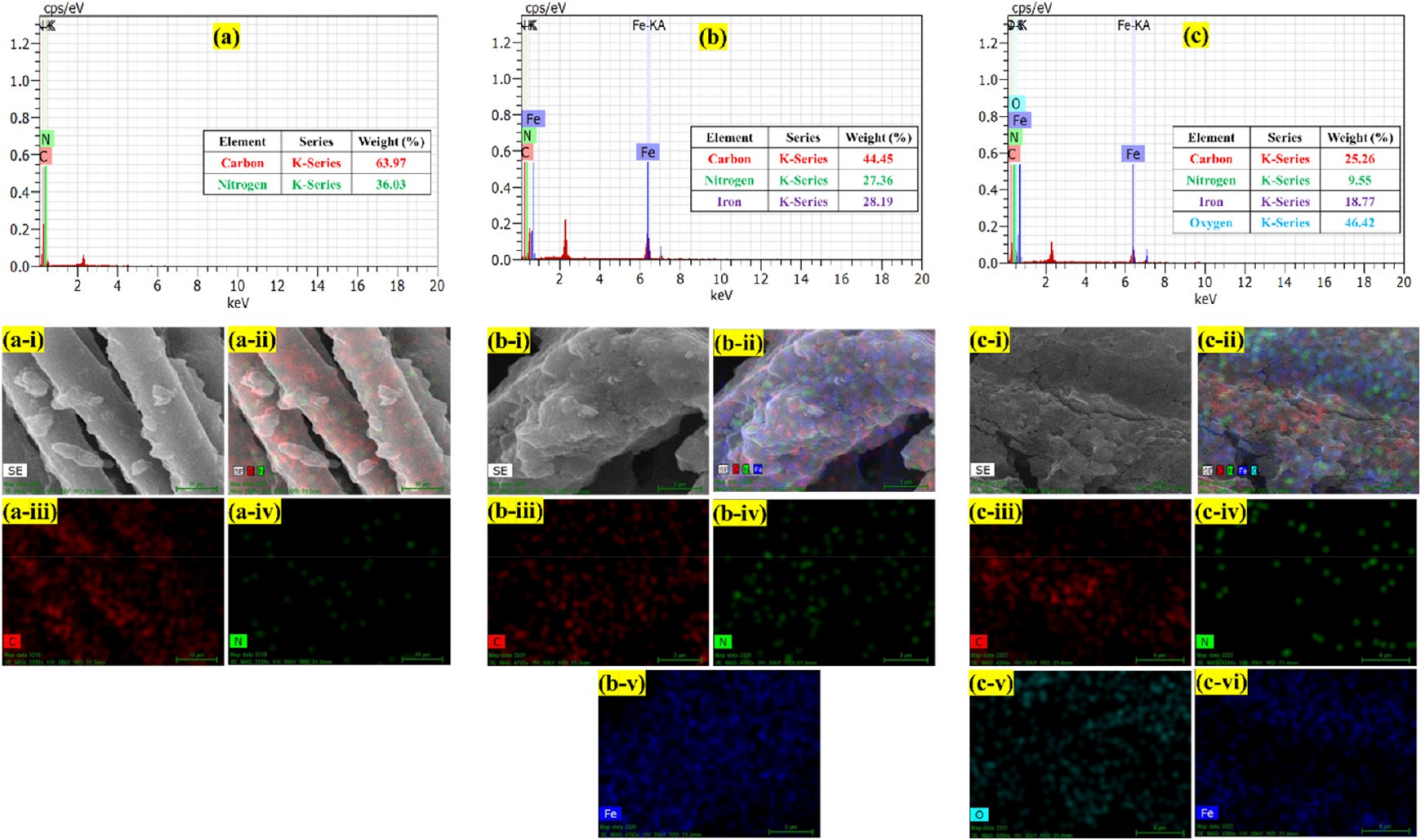
EDAX spectra of (a) PANI, (b) PANI-Fe, and (c) PANI-Fe-CQD, and the elemental analysis (a­(i-iv)) PANI, (b­(i-v)) PANI-Fe, and (c­(i-vi)) PANI-Fe-CQD.

#### 3.2.4 X-ray Photoelectron Spectroscopy (XPS)

The chemical composition and valence states of elements present in the synthesized PANI-Fe-CQD electrode were thoroughly examined by using XPS. The wide-scan survey spectrum, shown in Figure 6(a), reveals the successful incorporation of carbon (C), nitrogen (N), oxygen (O), and iron (Fe) within the electrode matrix. Quantitative elemental analysis (Figure 6(b)) indicates their respective atomic percentages as 65.93% for carbon, 5.83% for nitrogen, 14.34% for oxygen, and 13.89% for iron, confirming the multicomponent nature of the hybrid electrode material. The C 1s spectrum (Figure 6(c)) provides insights into the carbon bonding environments. A prominent peak observed at 284.48 eV is ascribed to C–C and C–H bonds,^39^ which are typical of the aromatic backbone of polyaniline (PANI). Additionally, the peak at 285.28 eV corresponds to C–N bonds, indicating successful nitrogen incorporation from PANI. A higher binding energy feature at 286.98 eV is assigned to carbon in carbonyl groups (C=O), reflecting the surface functionalization from CQDs and possibly oxidation during processing.^40^ The N 1s spectrum, depicted in Figure 6(d), further substantiates the nitrogen bonding states within the composite. The peak at 399.48 eV is attributed to imine-type nitrogen (=N-), while the signal at 400.78 eV is assigned to protonated amine nitrogen (-N^+^-H). These nitrogen functionalities are known to coordinate with transition metals, such as iron, forming strong Fe–N interactions.^41^ Such coordination is critical, as it enhances pseudocapacitive behavior by promoting efficient redox activity and improving charge transport dynamics in electrochemical applications. In Figure 6(e), the O 1s XPS spectrum exhibits a prominent peak at 531.78 eV, which is attributed to O–N bonding. This feature indicates a strong interaction between oxygen-containing functional groups and nitrogen species, likely originating from the nitrogen-doped carbon framework or the polymeric matrix. Such O–N bonds suggest that oxygen atoms from surface groups, particularly hydroxyl (−OH) and carboxyl (−COOH) moieties present on the CQDs, are chemically interacting with nitrogenous sites, potentially through hydrogen bonding, thus promoting structural integration of the composite. Additionally, a nearby peak at 531.66 eV is assigned to Fe–O bonding,^42^ confirming the presence of iron–oxygen linkages. This implies that oxygen functionalities on the CQD surface also coordinate with the iron species, thereby contributing to a stable and interconnected network within the composite. These dual interactions (O–N and Fe–O) underscore the active role of surface functional groups in mediating strong interfacial interactions, which can enhance electron transport pathways, improve structural stability, and ultimately lead to a superior electrochemical performance.

**Figure 6:**
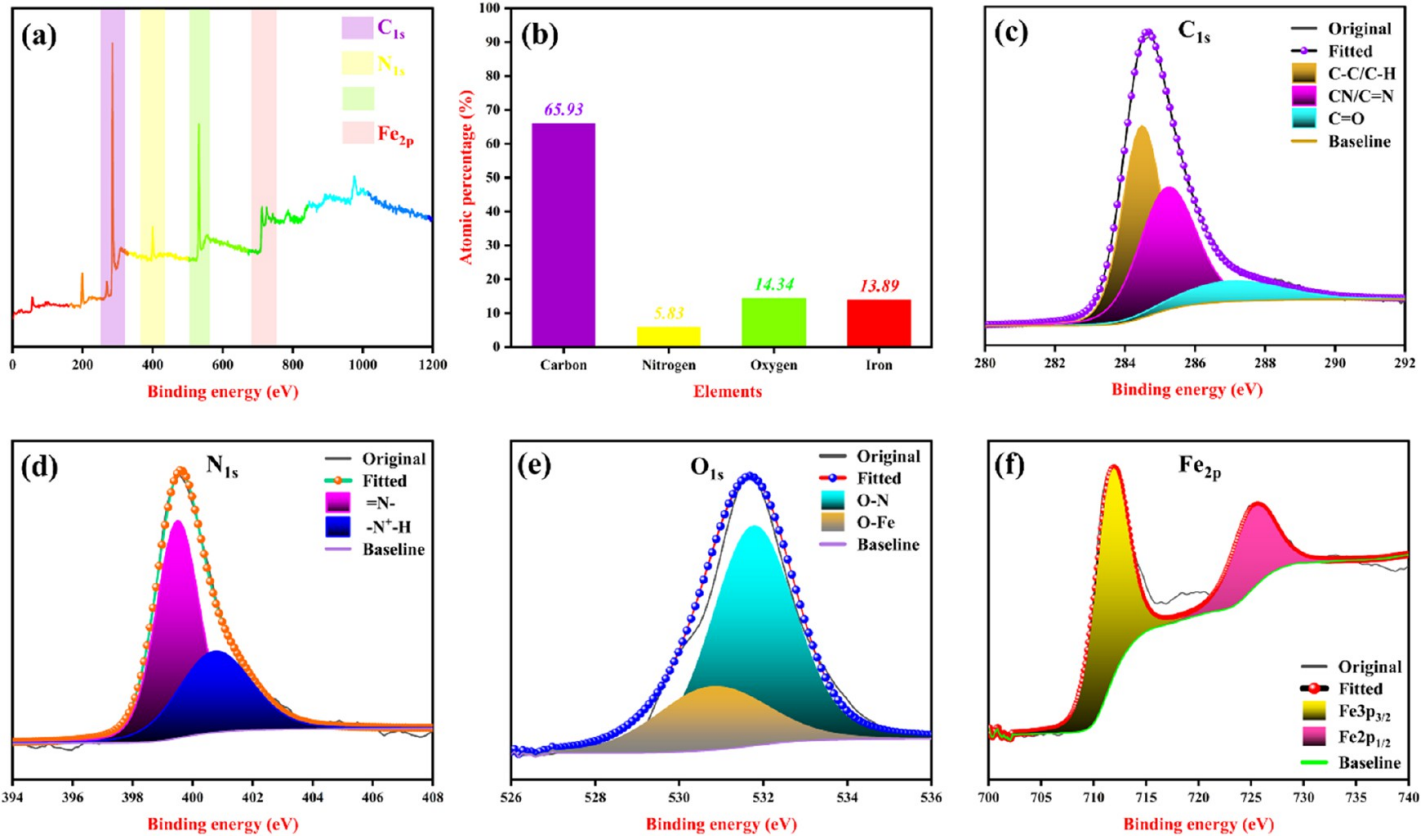
XPS deconvolution spectra of PANI-Fe-CQD (a) survey spectrum, (b) atomic percentage of elements, (c) C 1s spectra, (d) N 1s spectra, (e) O 1s spectra, and (f) Fe 2p spectra.

The Fe 2p XPS spectrum shown in Figure 6(f) reveals two well-defined peaks at 725.58 and 711.28 eV, attributed to the Fe 2p_1/2_ and Fe 2p_3/2_ spin–orbit components. These binding energies are characteristic of trivalent iron (Fe^3+^) species, confirming the oxidation state of iron in the composite.^43^ The presence of Fe^3+^ is particularly significant, as it can serve as a redox-active center during the electro-polymerization of PANI, and these Fe^3+^ ions facilitate the doping–dedoping process by participating in reversible redox reactions, which enhances charge storage capability, and the uniform distribution of Fe^3+^ throughout the PANI-Fe-CQD matrix improves electrical conductivity and electrochemical reversibility.

### 3.3 Supercapacitor Application

#### 3.3.1.1 Electrochemical Application-Three-Electrode System

The electrochemical performance of the fabricated flexible electrodes, PANI, PANI-Fe, and PANI-Fe-CQD, was evaluated using CV in a 0.5 M H_2_SO_4_ electrolyte. Figure 7(a) illustrates the CV curves of the fabricated electrodes recorded at a scan rate of 10 mV/s. The CV curve of the pristine PANI electrode displays a distinct oxidation peak at approximately 0.65 V, which corresponds to the redox transition characteristic of PANI between its different oxidation states (leucoemeraldine ↔ emeraldine ↔ pernigraniline).^44^ This confirms the pseudocapacitive nature of PANI arising from its electroactive nitrogen sites. In contrast, the bare carbon cloth (CC) substrate shows a nearly ideal rectangular CV profile, typical of electric double-layer capacitance (EDLC) behavior.^45^ This shape reflects capacitive ion adsorption/desorption at the electrode and electrolyte interface without involving any faradaic reactions. The absence of oxidation and reduction peaks in CC supports the fact that it lacks redox-active functional groups. However, upon deposition of PANI onto CC, the appearance of oxidation peaks confirms successful electro-polymerization and incorporation of nitrogen-containing groups, validating the presence of PANI on the CC surface. The PANI-Fe electrode demonstrates enhanced electrochemical activity, with two distinct oxidation peaks at approximately 0.38 and 0.63 V. These peaks are indicative of synergistic redox interactions between the nitrogen atoms from PANI and iron species incorporated into the electrode matrix. The presence of Fe is believed to facilitate electron transfer and introduce additional redox-active sites, thereby contributing to the improved charge storage capability. Notably, the anodic current response of PANI-Fe remains comparable to that of pure PANI, suggesting that the inclusion of Fe maintains or slightly improves the redox kinetics while introducing an additional oxidation process that may benefit the charge–discharge behavior.^46^ Further enhancement is observed in the PANI-Fe-CQD electrode, where the incorporation of CQDs leads to a significant increase in anodic current, from 5.84 mA (PANI-Fe) to 15.10 mA. The CQDs, rich in −OH and −COOH, not only improve the surface wettability and conductivity but also participate in charge transfer processes. This contributes to a higher electroactive surface area and more accessible redox sites, resulting in an improved faradaic response.

**Figure 7:**
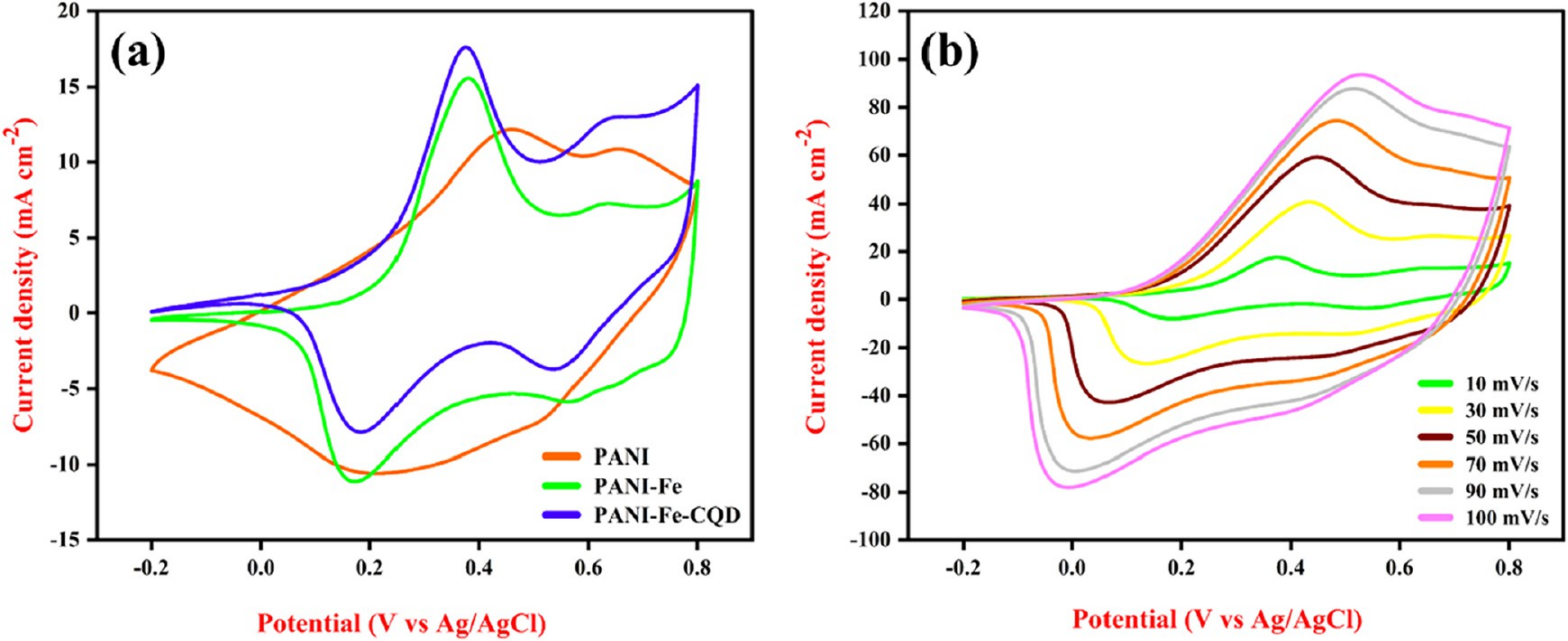
(a) CV curves of the fabricated PANI, PANI-Fe, and PANI-Fe-CQD at 10 mV/s and (b) different scan rate studies of PANI-Fe-CQD.

Figure 7(b) illustrates the CV profiles of the PANI-Fe-CQD electrode recorded at various scan rates ranging from 10 to 50 mV/s. As the scan rate increases, a noticeable enlargement in the enclosed area of the CV curves is observed, indicating enhanced capacitive behavior and greater charge storage capability. Additionally, upon increasing the scan rate, the oxidation and reduction peaks exhibit a progressive shift toward positive and negative potentials, respectively. This peak separation reflects faster redox kinetics and efficient ion transport within the electrode material, thereby demonstrating the electrode’s excellent rate capability and electrochemical reversibility. Similarly, for PANI, PANI-Fe, the different scan rate studies were performed and displayed in Figure S4.

The pseudocapacitive performance of the fabricated PANI-Fe-CQD polymerized flexible electrode was thoroughly analyzed using the Trasatti method, a well-established electrochemical technique used to distinguish between surface (outer) and diffusion-controlled (inner) charge storage contributions in supercapacitor electrodes.^47^ In this method, the total capacitance of the electrodes is first calculated at various scan rates using eq 1 and plotted accordingly. This total capacitance includes both the outer-surface-controlled processes and inner diffusion-controlled processes. Figure 8(a–c) present the total capacitance values obtained for the PANI, PANI-Fe, and PANI-Fe-CQD electrodes, respectively. The outer capacitance attributed to fast, nondiffusion-limited surface redox reactions is determined using eq 2, typically by extrapolating the capacitance to infinite scan rates. The corresponding data are shown in Figure 8(d–f). The inner capacitance, representing the slower, diffusion-limited processes that occur within the porous electrode or bulk material,^48^ is then calculated by subtracting the outer capacitance from the total capacitance, using eq 3, in Figure 8(g,h). PANI-Fe-CQD fabricated electrode delivers the total capacitance of 1321 mF/cm^2^, considered as 100%. Notably, 1286.8 mF/cm^2^ (97.41%) of this is attributed to inner (diffusion-controlled) capacitance, while only 34.2 mF/cm^2^ (2.59%) is due to outer (surface-controlled) capacitance indicating that the superior electrochemical performance of the PANI-Fe-CQD electrode is primarily governed by efficient ion diffusion within the electrode matrix, which facilitates the synergistic combination of PANI, Fe^3+^, and CQDs. PANI delivers a total capacitance of 287.35 mF/cm^2^, with a relatively lower inner capacitance of 157.94 F/cm^2^ (54.96%) and a considerable outer capacitance of 129.41 mF/cm^2^ (45.04%), suggesting a dominance of surface reactions over diffusion-based processes. PANI-Fe achieves a higher total capacitance of 1123.59 mF/cm^2^, with the inner capacitance accounting for 1091.19 mF/cm^2^ (97.11%) and the outer capacitance only 32.40 mF/cm^2^ (2.89%). This also points to a diffusion-dominated charge storage mechanism, but slightly lower than that of the PANI-Fe-CQD composite. The slope and *R* ^2^ values of the Trasatii plot are illustrated in Figure S5.

1 $$q^{*}\left(V\right)=K\frac{1}{V^{1/2}}+{q_{o}}^{*}$$

2 $$q^{*}{\left(V\right)}^{-1}=Kv^{1/2}+{q_{t}}^{*}$$

3 $${q_{t}}^{*}={q_{i}}^{*}+{q_{o}}^{*}$$

**Figure 8:**
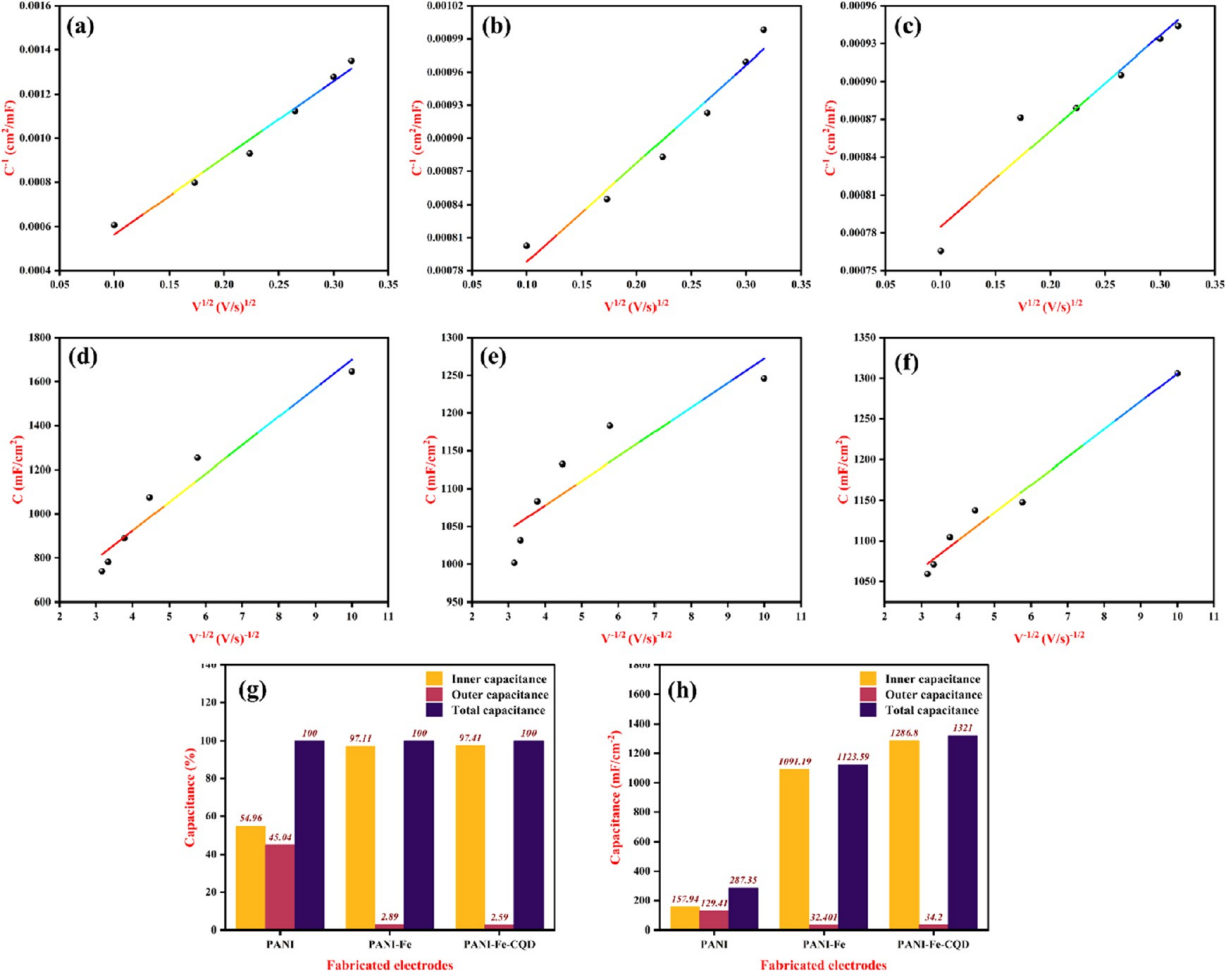
Trasatti plot: total capacitance calculated for (a) PANI, (b) PANI-Fe, and (c) PANI-Fe-CQD. Outer capacitance calculated for (d) PANI, (e) PANI-Fe, and (f) PANI-Fe-CQD. The (g) bar representation of total, outer, and inner capacitance and their (h) percentage of total, outer, and inner capacitance.

Figure 9(a–f) depict the analysis of the charge storage proportion in the PANI, PANI-Fe, and PANI-Fe-CQD flexible electrodes that was evaluated by the Dunn method, which effectively separates the total current response obtained from CV into capacitive (surface-controlled) and diffusion-controlled contributions. According to this method, the current at a given potential can be expressed as a combination of two terms by means of proportional to the scan rate (representing capacitive processes) and the other by proportional to the square root of the scan rate (associated with diffusion-limited ion transport), and indicates the charge stored in the electrodes under different scan rates. At a low scan rate of 10 mV/s, a significant portion of the current arises from diffusion-controlled processes, indicating that ions have ample time to penetrate the porous structure of the electrode and access internal redox-active sites (Figure 9(a–c)). However, as the scan rate increases to 100 mV/s (Figure 9(d–f)), the time available for ion diffusion decreases,^49^ limiting access to deeper sites, and the charge storage becomes increasingly dominated by capacitive processes occurring near the electrode surface. This shift enhances the surface reactivity and fast charge transfer characteristics of the materials, particularly in the PANI-Fe-CQD composite, which maintains a high capacitance contribution even at higher scan rates, and this behavior is indicative of excellent electrochemical kinetics and strong electrode/electrolyte interaction, enabling rapid ion migration and facilitating high-rate capability and cycling stability. Figure 9(g–i) displays the portions of capacitance and diffusion at different scan rates of the flexible electrode materials, and their partition is illustrated in Figure S6.

**Figure 9:**
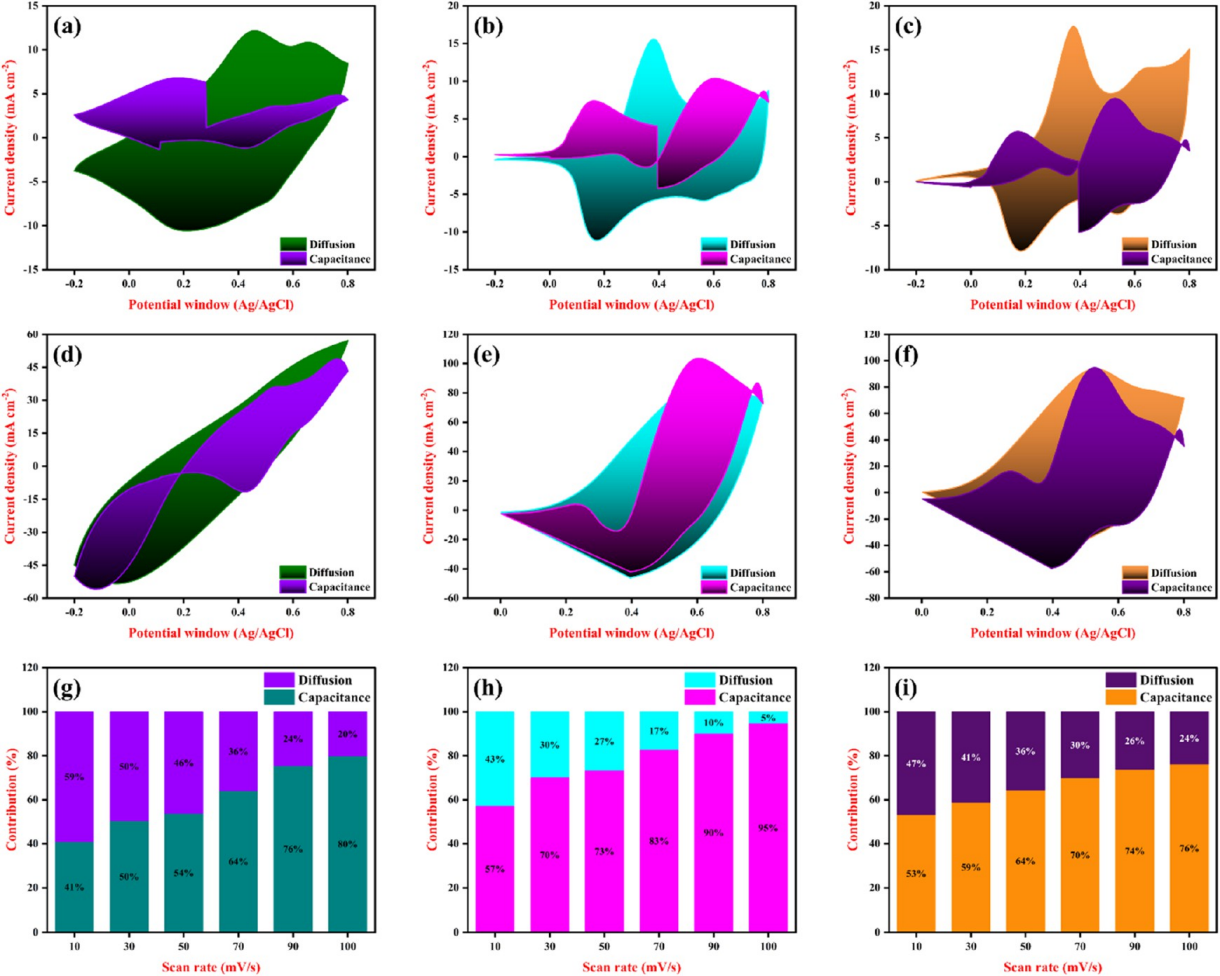
Dunn method: calculated from CV at 10 mV/s (a) PANI, (b) PANI-Fe, (c) PANI-Fe-CQD; calculated from CV at 100 mV/s (d) PANI, (e) PANI-Fe, (f) PANI-Fe-CQD. Bar representation of different scan rates from (10–100 mV/s), (g) PANI, (h) PANI-Fe, and (i) PANI-Fe-CQD.

Electrochemical impedance spectroscopy (EIS) is a crucial method for evaluating the resistive behavior of the fabricated electrodes. As shown in Figure S3­(a,b), the EIS analysis reveals that the solution resistance (*R*_s_) values for the PANI and PANI-Fe electrodes are 3.09 Ω and 2.07 Ω, respectively. Notably, the PANI-Fe-CQD composite exhibits the lowest Rs of 1.81 Ω, indicating enhanced electrical conductivity (Figure 10(a)). The EIS Nyquist plots for PANI-Fe and PANI-Fe-CQD display distinct semicircles in the high-frequency region, which are indicative of charge-transfer resistance at the electrode and electrolyte interface. The PANI electrode does not show a clear semicircle due to higher resistance and poorer charge-transfer characteristics. The reduced (*R*_s_) in the PANI-Fe-CQD electrode can be attributed to the synergistic effect of Fe incorporation and the conductive nature of CQDs, which facilitates more efficient charge transport and improved electrochemical performance.^50^ The electrochemical charge-storage behavior of the fabricated electrodes was evaluated through a GCD profile, which involves applying a constant current to the electrodes while recording their voltage response over time. From the obtained GCD curves, the charge-storage capability per unit area (areal capacitance) of the electrode was calculated using eq 4.^51^

4 $$C=\frac{I\times \Delta t}{\Delta v\times A}$$

Where *C* is the areal capacitance, Δ*t* is the discharge time, Δ*V* is the potential window during discharge, and A is the effective area of the electrode in cm^2^. Figure 10(b) presents the GCD curves recorded at a current density of 1 mA cm^–2^ for the flexible electrodes. The areal capacitances calculated for the PANI, PANI-Fe, and PANI-Fe-CQD electrodes were 643 mF/cm^2^, 831.25 mF/cm^2^, and 1212.5 mF/cm^2,^ respectively, at 1 mA/cm^2^ (Figure 10(c)). Among these, the PANI-Fe-CQD electrode exhibited the highest areal capacitance. This superior performance is attributed to the synergistic effect of EDLC from the CQDs and the pseudocapacitive contributions from both Fe and PANI. The presence of Fe^3+^ enhances redox activity, while the CQDs improve electronic conductivity and surface area, thereby facilitating more efficient charge transfer and greater energy storage capacity. The GCD performance of the PANI-Fe-CQD electrode was evaluated at varying current densities ranging from 1 to 5 mA/cm^2^ (Figure 10(d)). The corresponding areal capacitances were measured to be 1212.5 mF/cm^2^, 1125 mF/cm^2^, 1050 mF/cm^2^, 1000 mF/cm^–2^, 937.5 mF/cm^2^, and 915.3 mF/cm^2^, respectively, with a decreasing capacitance with increasing current density was observed this decrease in areal capacitance can be attributed to the limited time available for effective ion diffusion and charge storage at higher currents. At lower current densities, the electrode material facilitates more efficient faradaic redox reactions over the entire active surface area, thereby contributing to higher capacitance. Meanwhile, the current density increases, the rapid charge–discharge process restricts ion transport and access to internal active sites, resulting in reduced utilization of the electroactive surface and lower capacitance.

**Figure 10:**
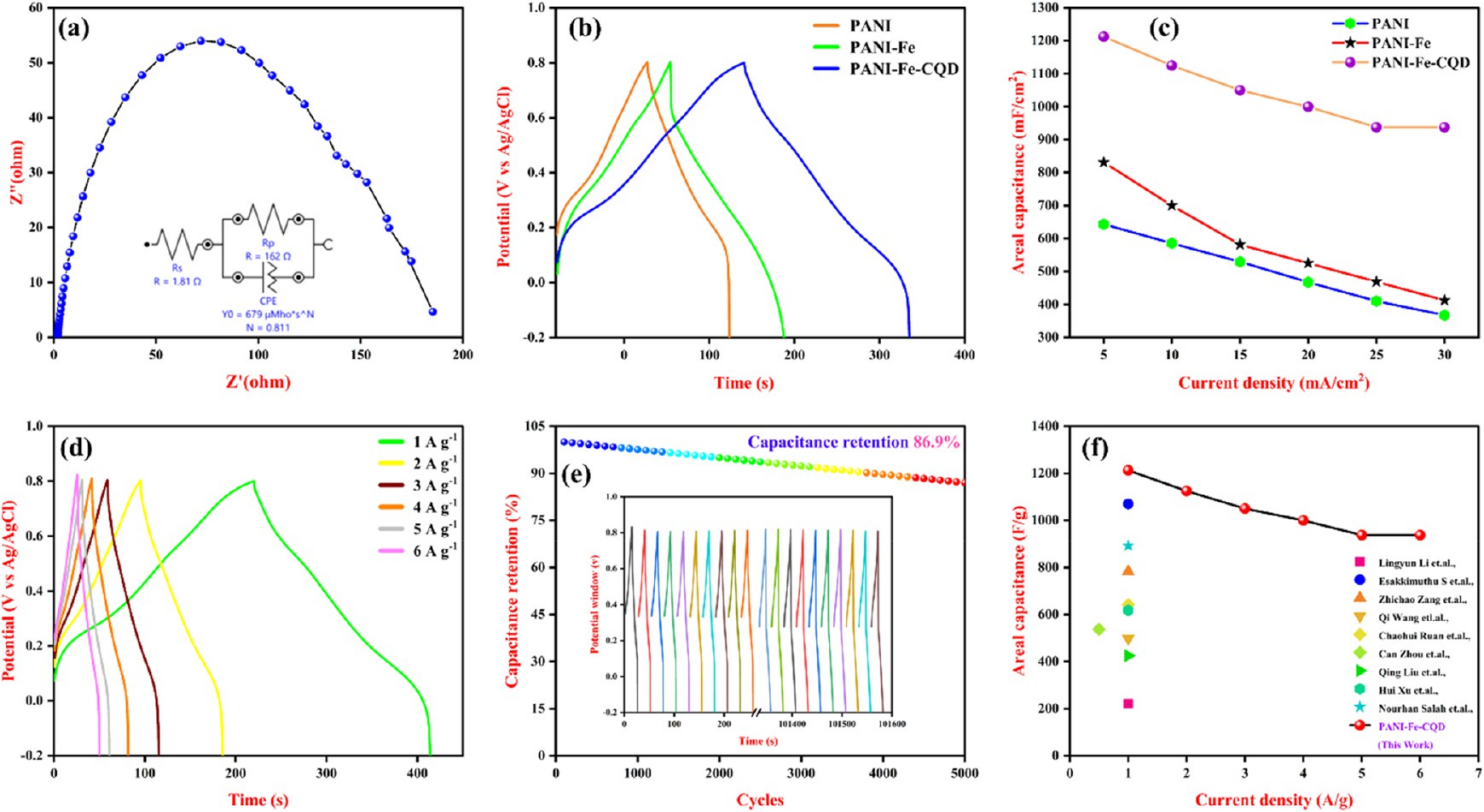
(a) EIS studies of PANI-Fe-CQD, (b) GCD comparison of fabricated electrodes, (c) areal capacitance graph for the fabricated flexible electrodes, (d) GCD of PANI-Fe-CQD at different current densities, (e) cyclic stability of PANI-Fe-CQD (inset: first and last 10 cycles), and (f) comparison of previously reported literature.

The long-term electrochemical stability of the fabricated PANI-Fe-CQD electrode was assessed through GCD cycling over 5000 continuous cycles, as shown in Figure 10(e). As a result, the electrode retained 86.9% of its capacitance, indicating a lower decrease in performance. This high capacitance retention exhibits excellent cycling stability and structural robustness of the PANI-Fe-CQD flexible electrode. The minimal loss of capacitance demonstrates that the electrode material effectively withstands the mechanical and electrochemical stresses associated with repeated ion intercalation and redox reactions. The three-electrode performance of the fabricated PANI-Fe-CQD was compared with the previously reported articles, as illustrated in Figure 10(f) and stated in Table 1.

**Table 1:** Comparison of PANI-Fe-CQD with the Previously Reported Literature

S.No	PANI and CQD-based composites	CQD source	electrolyte	current density	capacitance	refs
1	PANI/CQD	citric acid	1 M H_2_SO_4_	1 A g^–1^	222 F g^–1^	^52^
2	CuS/C@PANI	-	3 M KCl	1 A g^–1^	425.53 F g^–1^	^53^
3	PANI/ZnS QD	-	1 M H_2_SO_4_	1 A g^–1^	893 F g^–1^	^54^
4	CQD/PANI	electrooxidation of graphene	1 M H_2_SO_4_	1 A g^–1^	738 F g^–1^	^55^
5	PANI-Cu	-	0.5 M H_2_SO_4_	1 A g^–1^	618Fg^–1^	^56^
6	PPy/CQD	citric acid	1 M Na_2_SO_4_	0.5 A/g	537.9 F/g	^22^
7	PANI/N-CQD	citric acid	1 M H_2_SO_4_	1 A g^–1^	498 F g^–1^	^57^
8	PANI-CQD-Cu	ascorbic acid	1 M H_2_SO_4_	1 A g^–1^	1070 F g^–1^	^21^
9	PANI/Fe	-	1 M H_2_SO_4_	1 A g^–1^	642 F g^–1^	^58^
**10**	**PANI-Fe-CQD**	***B. flabellifer* (Biomass source)**	**0.5 M H**_**2**_ **SO**_**4**_	**5**mA/cm^**2**^ **or 1**A/g	**1212.5 mF/cm** ^**2**^ **or 1212 F/g**	**this work**

#### 3.3.1.2 Electrochemical Application: Two-Electrode System

The operational potential window of the fabricated two-electrode supercapacitor device (AC//PVA-H_2_SO_4_//PANI-Fe-CQD) was derived from the individual electrochemical behavior of the constituent electrodes in a three-electrode system. In this two-electrode setup, acetylene carbon (AC) was employed as the cathode (negative terminal), exhibiting a stable potential range from −1 to 0 V. Whereas the fabricated flexible PANI-Fe-CQD composite plays as the anode (positive terminal), with these electrode setup an extended potential range from −2.0 V to +0.8 V was observed with quasi-solid-state PVA/H_2_SO_4_ gel as electrolyte^55^ at 50 mV/s, facilitating ionic conduction between the electrodes as depicted in Figure 11(a).

**Figure 11:**
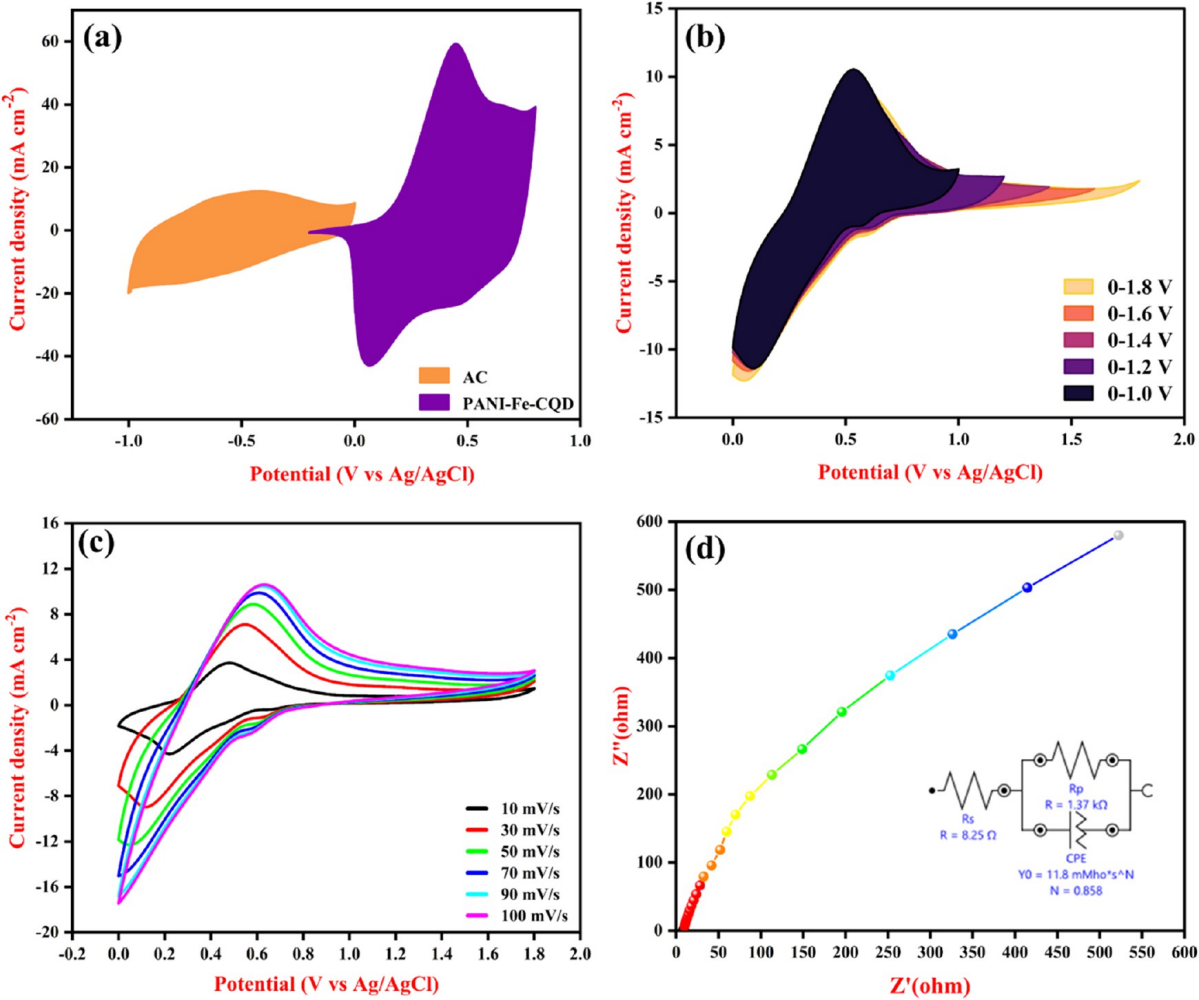
(a) Three-electrode system CV of AC and PANI-Fe-CQD in 50 mV/s, (b) CV different potential window analysis, (c) different scan rate studies of optimized potential window, and (d) EIS studies of the AC//PVA-H_2_SO_4_//PANI-Fe-CQD device.

The CV experiments were performed to study the working potential window of AC//PVA-H_2_SO_4_//PANI-Fe-CQD at scan rates of 10 mV/s and 100 mV/s across different potential windows from 0 to 1 V to 0–1.8 V, as shown in Figures 11(b) and S7­(a), respectively. The results reveal that the potential window is extended from 0 to 0.8 V to 0–1.8 V at both the scan rates, the area enclosed by the CV curves progressively increases, and this expansion in CV area directly correlates with enhanced charge storage capability, indicating a higher capacitance. Figure 11(c) represents the CV curves at different scan rates within the optimized potential window of 0–1.8 V. As the scan rate increases, both oxidation and reduction peak currents shift toward more positive and negative values, insights transition from purely diffusion-controlled charge storage mechanisms to more surface-dominated redox processes. The broadened peak separation and higher current response at faster scan rates suggest enhanced kinetics and improved electrode–electrolyte interaction at the electrode surface. Moreover, similar CV analyses performed for intermediate voltage ranges 0–1.0 V to 0–1.6 V are displayed in Figure S7­(b–d), respectively. Figure 11(d) exhibits the EIS studies of the AC//PVA-H_2_SO_4_//PANI-Fe-CQD device, which possesses a relatively low solution resistance (*R*_s_) of 8.25 Ω, and this low Rs value favors efficient ionic conductivity within the quasi-solid-state PVA-H_2_SO_4_ electrolyte and minimal resistance at the electrode/electrolyte interface. A lower resistance is suitable for rapid ion transport and reduced internal energy losses, which contribute to better charge–discharge performance.^59^ The semicircular region (Warburg impedance) in the high-frequency Nyquist plot reflects the charge transfer resistance (Rct), and the nearly vertical line in the low-frequency region is suitable for ideal capacitive behavior with good ion diffusion. Figure S8 reveals the CV of the fabricated AC//PVA-H_2_SO_4_//PANI-Fe-CQD electrode in the flat and bent positions at 10 mV/s. The result reveals that in both positions, there is no change in the CV curves observed, thus indicating the flexible nature of the fabricated electrode.

Figure 12(a) illustrates the GCD curves of the AC//PVA-H_2_SO_4_//PANI-Fe-CQD device measured at various potential windows at a fixed current density of 1 A g^–1^. As the applied potential window increases from 0 to 1.0 V to 0–1.8 V, the charge–discharge profiles show a corresponding increase in the discharge time, indicating enhanced charge storage capacity. The areal capacitance values calculated from these GCD curves progressively rise with increasing voltage, 103 mF/cm^2^ at 0–1.0 V, 106 mF/cm^2^ at 0–1.2 V, 110 mF/cm^2^ at 0–1.4 V, 113 mF/cm^2^ at 0–1.6 V, and reaching a maximum of 119 mF/cm^2^ at 0–1.8 V, and this indicates that the device exhibits its highest areal capacitance at the expanded potential window of 1.8 V and enables the storage of energy as shown in Figure 12 (b). The GCD analysis was carried out at varying current densities ranging from 1 A g^–1^ to 5 A g^–1^ for the potential window 0–1.6 V, as shown in Figure 12(c). Similarly, for the other windows, the GCD was analyzed and is illustrated in Figure S9. The areal capacitance gradually decreases with increasing current density was noted which is due to the limited time available for ions to diffuse and access all active sites within the electrode material at higher current rates, resulting in lower charge storage (Figure 12(d)). With these capacitance values, the energy density and power density are calculated using eqs 5 and 6, respectively.

5 $$E=\frac{C\times {\left(\Delta v\right)}^{2}}{7.2}$$

6 $$P=\frac{E\times \Delta t}{3.6}$$

Where *E* is the energy density, and *C* is the areal capacitance. (Δ*v*)^2^ is the square of the potential difference, and Δ*t* is the difference in time. The fabricated AC//PVA-H_2_SO_4_//PANI-Fe-CQD supercapacitor exhibits a maximum energy density of 53.25 μWh/cm^2^ at a corresponding power density of 0.9 mW/cm^2^, demonstrating excellent energy storage capability as displayed in the Ragone plot (Figure 12(f)), and a comparative analysis with previously reported literature were analyzed and listed in Table 2, which is clearly showing the superior performance of the current device. The long-term cyclic performance of the fabricated electrode was examined with the cyclic stability over 5000 GCD cycles, and the device exhibits capacitance retention over 91%, as illustrated in Figure 12(e). This results in the fabrication of PANI-Fe-CQD as a potent material for supercapacitor application.

**Figure 12:**
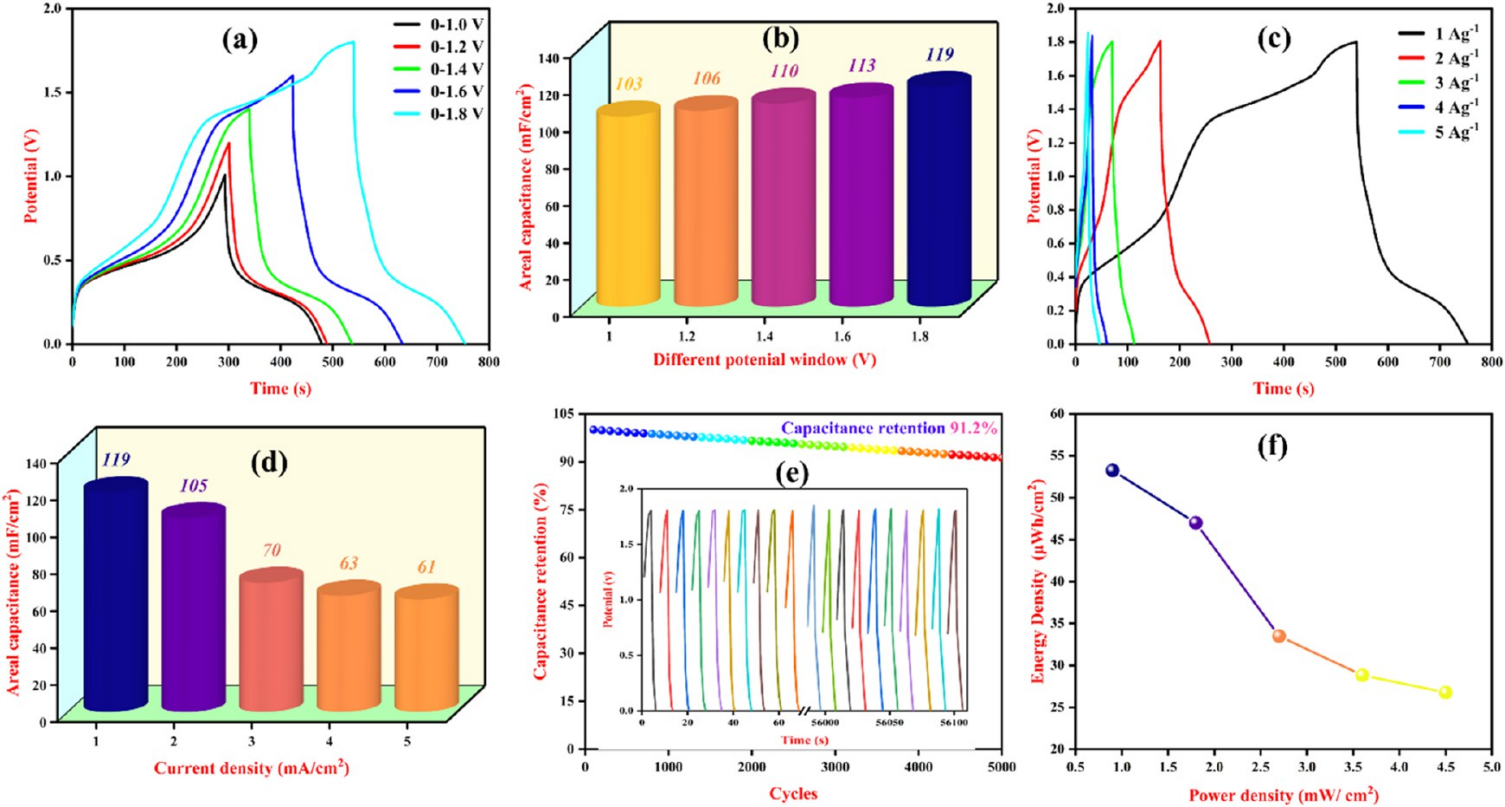
(a) GCD analysis on different potential windows, (b) bar representation of areal capacitance of different potential window, (c) GCD analysis of 1.6 V potential windows in different current densities, (d) bar representation of areal capacitance in 1.6 V potential window, (e) cyclic stability (inset: first and last 10 cycles), and (f) ragone plot for AC//PVA-H_2_SO_4_//PANI-Fe-CQD.

**Table 2:** Comparison of AC//PVA-H_2_SO_4_//PANI-Fe-CQD with Previously Reported Literature

S. No	symmetric/asymmetric	fabricated materials	energy density	power density	refs
1	symmetric	PPy/CQD	0.065 mWh/cm^2^	0.036 mW cm^–2^	^22^
2	asymmetric	PANI/CQD	0.051 mWh cm^–2^	48.4 mW cm^–2^	^20^
3	symmetric	PANI-CQD	33.8 mWh cm^–2^	0.3 mW cm^–2^	^55^
4	asymmetric	PANI-CQD-Cu	23.10 mWhcm^–2^	0.978 mW cm^–2^	^21^
**5**	**asymmetric**	**PANI-Fe-CQD**	**53.25 m Wh/cm** ^**2**^	**0.9 mW/cm^2^**	**this work**

#### 3.3.1.3 LED Light Illumination Experiment

The charge–discharge characteristics of the fabricated electrodes were experimentally investigated. This setup assembled a series of cathode and anode materials in a sandwich configuration, with a PVA-H_2_SO_4_ gel acting as a solid-state quasi-electrolyte. The assembled device was charged using an HW battery for 60 s. Upon disconnecting the power source, a discharge and a decrease in the LED glow were observed after 340 s. During this period, a gradual dimming of a connected LED was observed, indicating a progressive decrease in stored energy. This discharge behavior, including the LED brightness, is depicted in Figure 13 and Video 1.

**Figure 13:**
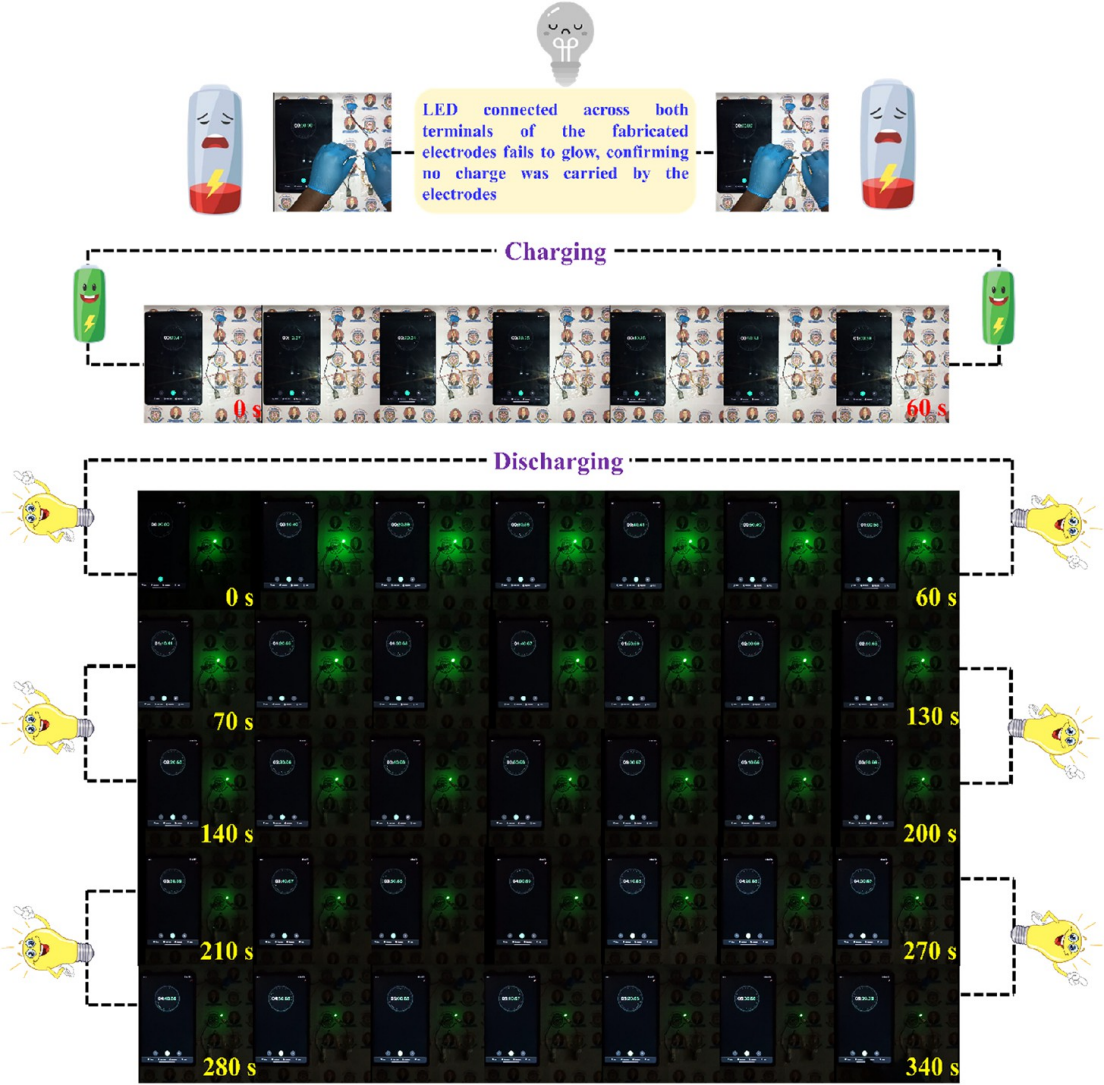
LED light illumination experiment.

## 4 Conclusion

This work shows the successful synthesis of biomass-derived CQDs from *B. flabellifer* through a simple one-pot hydrothermal method. The optical characterization revealed distinct π-π* and n-π* transitions at 203 and 310 nm, respectively. With this, the excitation-dependent fluorescence behavior confirms the promising photophysical properties of the CQDs. Structural and morphological analyses, including FT-IR and TEM, verified the presence of functional groups and a uniform spherical morphology with an average particle size of 5.3 nm. The CQDs were further integrated into an electropolymerized PANI-Fe-CQD composite electrode. XRD and Raman spectroscopy evidenced successful doping of Fe and CQDs, as indicated by peak shifts, broadening of the (002) plane, and a decrease in the *I*_D_/*I*_G_ ratio, suggesting an improved structural interaction and electronic conductivity. SEM provided clear morphological validation of Fe and CQD incorporation into the carbon cloth matrix, while EDAX and XPS analyses confirmed the elemental composition and oxidation states. The electrochemical studies using a three-electrode setup show superior capacitive performance for the PANI-Fe-CQD electrode, achieving a remarkable areal capacitance of 1212.5 mF/cm^2^ and excellent cycling stability with 89.6% retention after 5000 cycles. Furthermore, an asymmetric supercapacitor device fabricated using AC//PVA-H_2_SO_4_//PANI-Fe-CQD exhibits an impressive energy density of 53.25 μWh/cm^2^ at a power density of 0.9 mW/cm^2^, along with a 91.2% capacitance retention over 5000 cycles. Finally, the overall performance of the PANI-Fe-CQD was experimentally investigated by the series setup using LED illumination with a discharge time over 340 s. The overall results show the significant potential of biomass-derived CQDs, particularly in conjunction with conducting polymers and metal doping, as a high-performance flexible electrode for next-generation energy storage and flexible supercapacitor devices.

## Supplementary Material





## Data Availability

The data sets used or analyzed during the current study are available from the corresponding author upon reasonable request.

## Notes

Physical and electrochemical characterization, fabrication of electrode materials, TEM particle size, TEM morphology, cyclic voltammetry, galvanostatic charge–discharge, and electrochemical impedance studies (PDF)
PANI-Fe-CQD (Vidoe S1) (MP4)
