# Supplementary material for: Fabrication of Electropolymerized Binder-Free Flexible Electrode of PANI-Fe-Doped Borassus flabellifer -Biomass-Derived (0D)-Carbon Quantum Dots for High-Performance Asymmetric Supercapacitors
Source: ACS Omega. 2025 Dec 2;10(49):60270–86. doi: 10.1021/acsomega.5c06469 (PMC12713462; doi:10.1021/acsomega.5c06469)
Supplement: Supplementary file 1 [file ao5c06469_si_001.pdf]

Supplementary information

Fabrication of Electropolymerized Binder-Free Flexible Electrode of PANI-Fe Doped *Borassus flabellifer* - Biomass Derived (0D)-Carbon Quantum Dots for High-Performance Asymmetric Supercapacitors

Nithesh Kumar Krishnan[1], Esakkimuthu Shanmugasundaram[2], Harini Bhagyaraj[1], Kannan Vellaisamy[1], Amos Ravi[3], As'ad Ibrahim,[4] Na'il Saleh[4]*, Stalin Thambusamy[1]*

[1]*Department of Industrial Chemistry, Alagappa University, Karaikudi - 630 003, Tamil Nadu, India.*

[2] *Department of Chemistry, Thiagarajar College of Engineering, Madurai- 625015, Tamil Nadu, India.*

[3]*Department of Materials Science, Central University of Tamil Nadu, Thiruvarur - 610 005, Tamil Nadu, India.*

[4]*Department of Chemistry, College of Science, United Arab Emirates University, P.O. Box 15551, Al Ain, United Arab Emirates.*

Corresponding authors e-mail:* ***Stalin Thambusamy - *stalin.t@alagappauniversity.ac.in.

***Na'il Saleh -** n.saleh@uaeu.ac.ae.

1. Experimental section

1.1. Materials required

Borassus flabelliformis was collected from Palm tree waste. Carbon Cloth (CC), Aniline ($C_6H_5NH_2$), Sulfuric acid (H_2SO_4), Ferrous sulphate (Fe_2SO_4), and Polyvinyl alcohol (PVA) were obtained from SRL Chemicals Private Ltd. Distilled water was used in all the experiments, and it was obtained from the MilliQ pore system.

1.2. Characterization techniques

The synthesized carbon quantum dots were preliminarily analyzed by a UV-Vis spectrometer (JASCO-600V) with a wavelength of 200nm- 800nm, and a Fluorescence spectrometer (JASCO FP-8200) with excitation monitored from the UV-Vis spectrometer. Fourier Transform-Infrared Red (FT-IR) analysis was performed using the FT-IR-JASCO-4600, in Attenuated Total Reflectance (ATR) mode for the liquid samples with the wavelength of 4000 cm^{-1} to 400 cm^{-1}. Morphology lattice points were examined with the help of the JEOL-2100+ High-Resolution Transmission Electron Microscope (HR-TEM) with an accelerating voltage of 200 kV in the resolution point of 0.194nm.

The fabricated flexible supercapacitor electrodes were characterized, and the XRD patterns were analyzed with an HT-Powder X-ray Diffractometer (XRD) with HTK 1200N-Bruker D8 Advance. The disorder in the structure was analyzed with an Imaging Spectrograph STR 500 mm Focal Length Laser Raman spectrometer with a flat field of 27 mm(W) x 14 mm(H) and resolution of 1 / 0.6cm^{-1}/pixel. The Morphology was examined using Carl Zeiss EVO 18 Microscopy GmbH, Germany, with a resolution of 3 nm and a magnification from 1x to 1000000x in High vacuum and variable Pressure was fitted with EDAX. The functional groups were examined with PHI - VERSAPROBE III – X-ray Photoelectron Spectroscopy, Monochromatic X-ray Beam (15µm), and the electrochemical analysis was performed using Metrohm electrochemical analyzer MULTIAUTOLAB-M204 (NOVA software).

1.3. Electrochemical analysis

The electrochemical analysis was examined with the three-electrode setup where the fabricated PANI, PANI-Fe, and PANI-Fe-CQD act as a working electrode, Ag/AgCl and platinum wire as reference and counter electrodes, respectively 0.5 M H_2SO_4 used as electrolyte to study the Cyclic

voltammetry (CV), Galvanostatic charge discharge (GCD), and Electrochemical impedance (EIS) characterizations were analyzed. In a two-electrode the fabricated PANI-Fe-CQD serves as a positive electrode, Acetylene carbon serves as a negative electrode, and the cellulose filter paper serves as a separator. For preparing the PVA-KOH gel electrolyte, 1 g of PVA was dissolved in 20 mL of deionized water and heated with continuous stirring until a clear gel was formed. About 0.5 M H_2SO_4 was added to the solution. The fabricated electrodes were then immersed in the prepared gel and aligned face-to-face to assemble the symmetric supercapacitor device (AC//PVA-H_2SO_4//PANI-Fe-CQD).

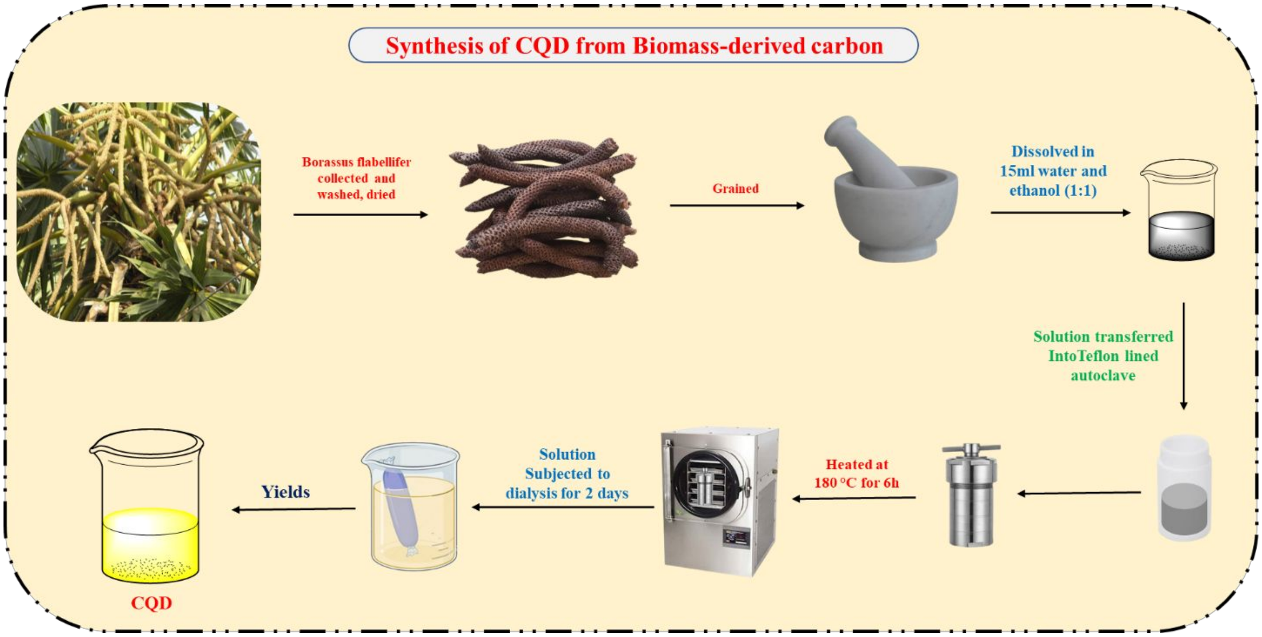

Scheme S1. Synthesis of CQDs from Borassus flabellifer**.**

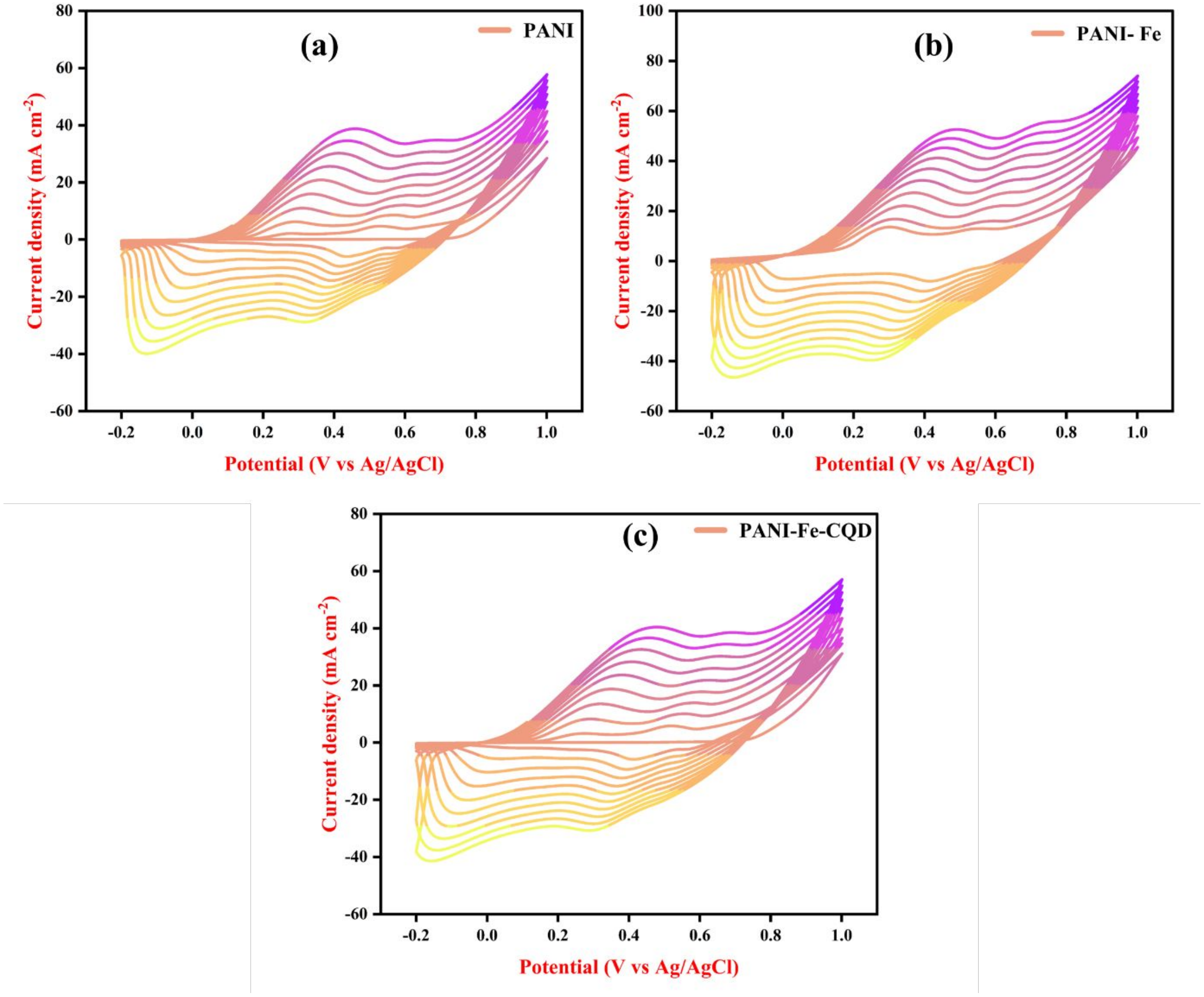

Figure S1. Electro polymerization curve to the (a) PANI, (b) PANI-Fe (c) PANI-Fe-CQD.

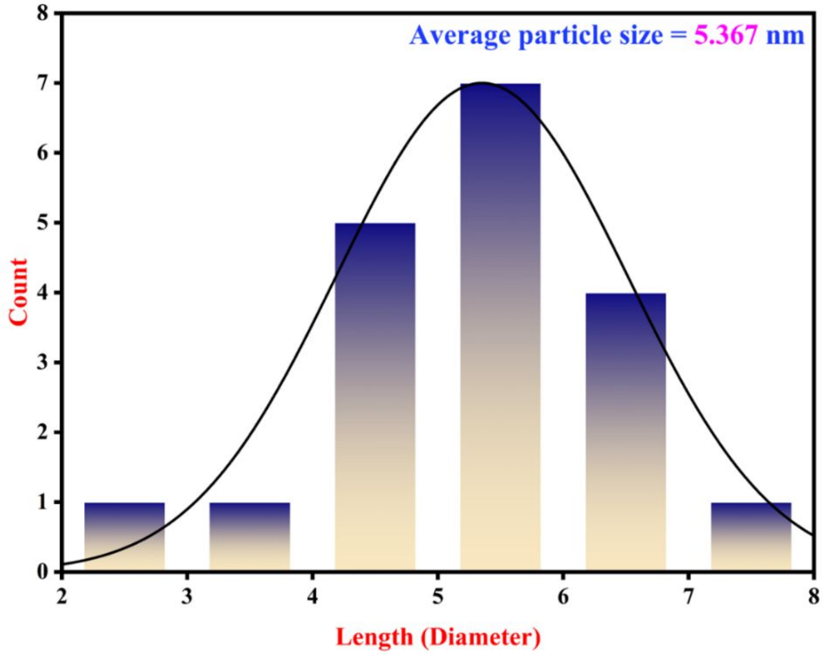

Figure S2. TEM particle size analysis of CQD.

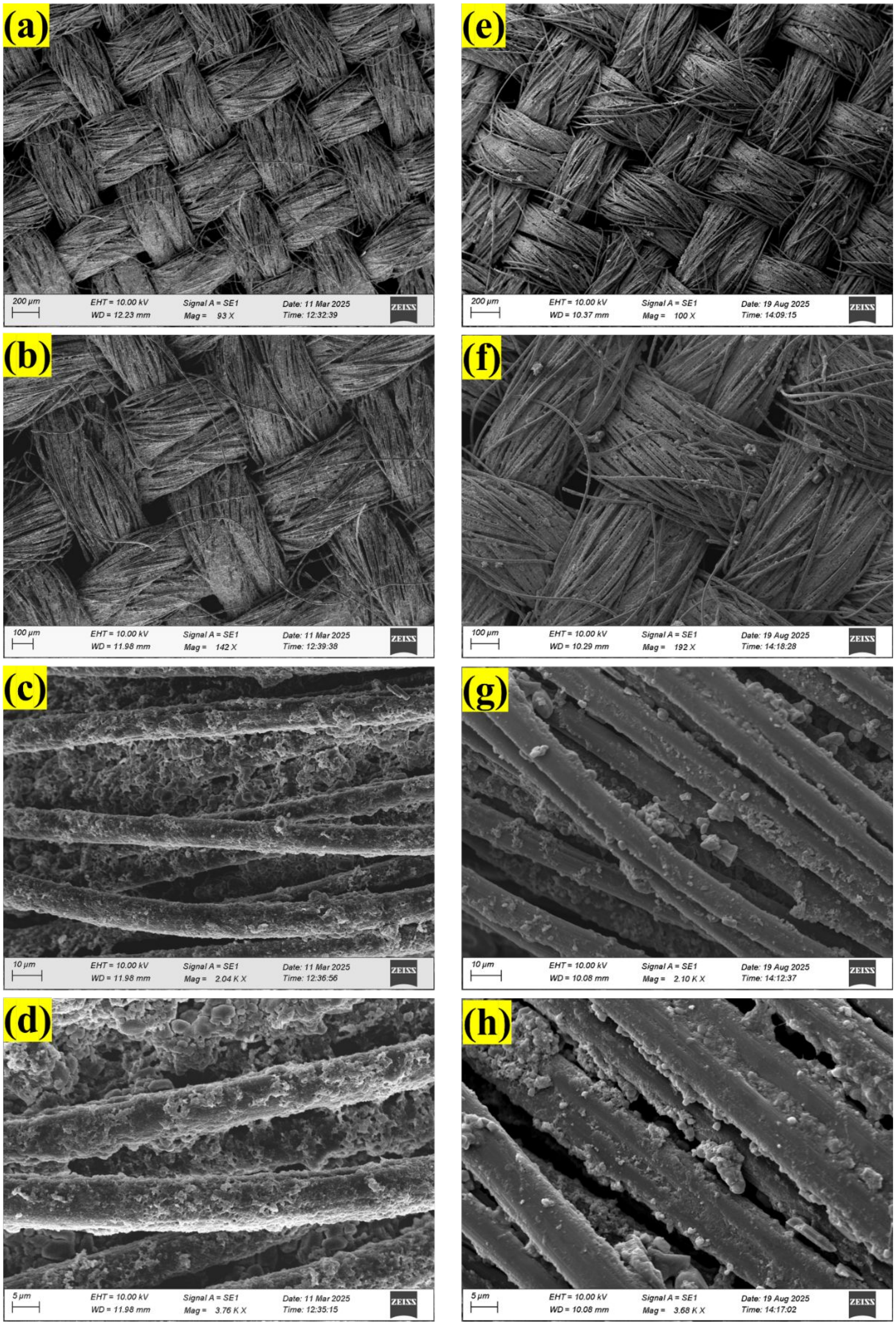

Figure S3. SEM morphology analysis of PANI-Fe-CQD (a-d) before and (e-h) after electrochemical analysis.

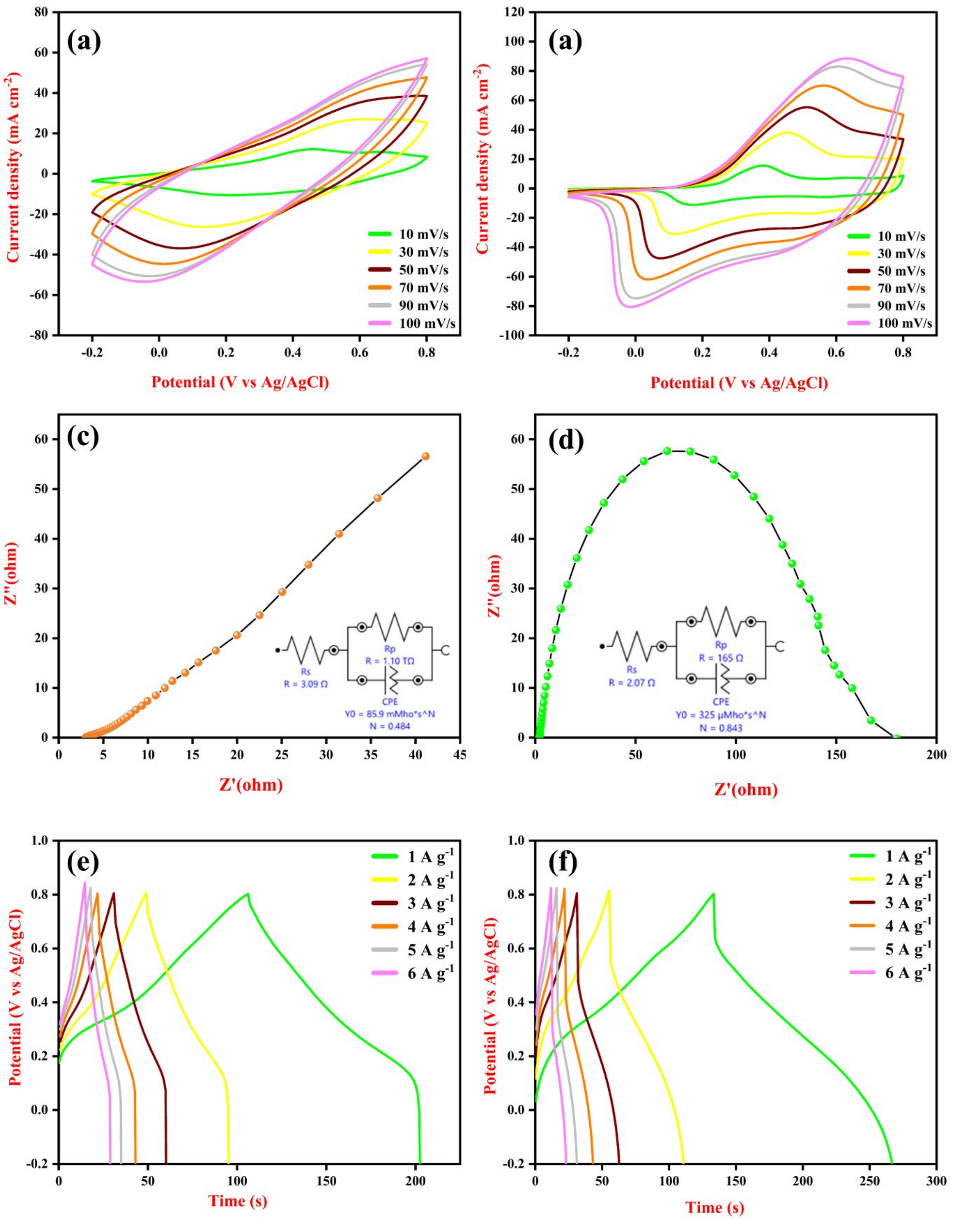
(a)
Current density (mA cm-2)
Potential (V vs Ag/AgCl)
10 mV/s
30 mV/s
50 mV/s
70 mV/s
90 mV/s
100 mV/s
(a)
(c)
Z''(ohm)
Z'(ohm)
Rs
R = 3.09 Ω
Rp
R = 1.10 TΩ
CPE
Y0 = 85.9 mMho*s^N
N = 0.484
(d)
Rs
R = 2.07 Ω
Rp
R = 165 Ω
CPE
Y0 = 325 μMho*s^N
N = 0.843
(e)
(f)
Potential (V vs Ag/AgCl)
Time (s)
1 A g-1
2 A g-1
3 A g-1
4 A g-1
5 A g-1
6 A g-1

Figure S4. The CV different scan rate studies of (a) PANI, (b) PANI-Fe, EIS studies of (c) PANI, (d) PANI-Fe, and the GCD studies with different current densities (e) PANI and (f) PANI-Fe.

PANI-Total capacitance

Equation	y = a + b*x
Plot	B
Weight	No Weighting
Intercept	2.1633E-4 ± 5.96529E-5
Slope	0.00348 ± 2.46986E-4
Residual Sum of Squares	8.2222E-9
Pearson's r	0.99007
R-Square (COD)	0.98024
Adj. R-Square	0.9753

PANI-Fe-Total capacitance

Equation	y = a + b*x
Plot	B
Weight	No Weighting
Intercept	6.99072E-4 ± 2.0402E-5
Slope	8.90686E-4 ± 8.44725E-5
Residual Sum of Squares	9.61772E-10
Pearson's r	0.98248
R-Square (COD)	0.96527
Adj. R-Square	0.95659

PANI-Fe-CQD-Total capacitance

Equation	y = a + b*x
Plot	B
Weight	No Weighting
Intercept	7.09278E-4 ± 2.43853E-5
Slope	7.57324E-4 ± 1.00965E-4
Residual Sum of Squares	1.37398E-9
Pearson's r	0.96624
R-Square (COD)	0.93362
Adj. R-Square	0.91703

PANI-Outer capacitance

Equation	y = a + b*x
Plot	D
Weight	No Weighting
Intercept	405.98628 ± 83.93611
Slope	129.41772 ± 14.96593
Residual Sum of Squares	29991.70342
Pearson's r	0.97428
R-Square (COD)	0.94923
Adj. R-Square	0.93653

PANI-Fe-Outer capacitance

Equation	y = a + b*x
Plot	D
Weight	No Weighting
Intercept	948.23762 ± 42.54085
Slope	32.40107 ± 7.5851
Residual Sum of Squares	7703.99217
Pearson's r	0.90565
R-Square (COD)	0.8202
Adj. R-Square	0.77525

PANI-Fe-CQD Outer capacitance

Equation	y = a + b*x
Plot	D
Weight	No Weighting
Intercept	963.76619 ± 15.01265
Slope	34.19766 ± 2.67678
Residual Sum of Squares	959.4401
Pearson's r	0.98797
R-Square (COD)	0.97608
Adj. R-Square	0.9701

Figure S5. The slope values of the Trasatii plot of PANI, PANI-Fe, and PANI-Fe-CQD.

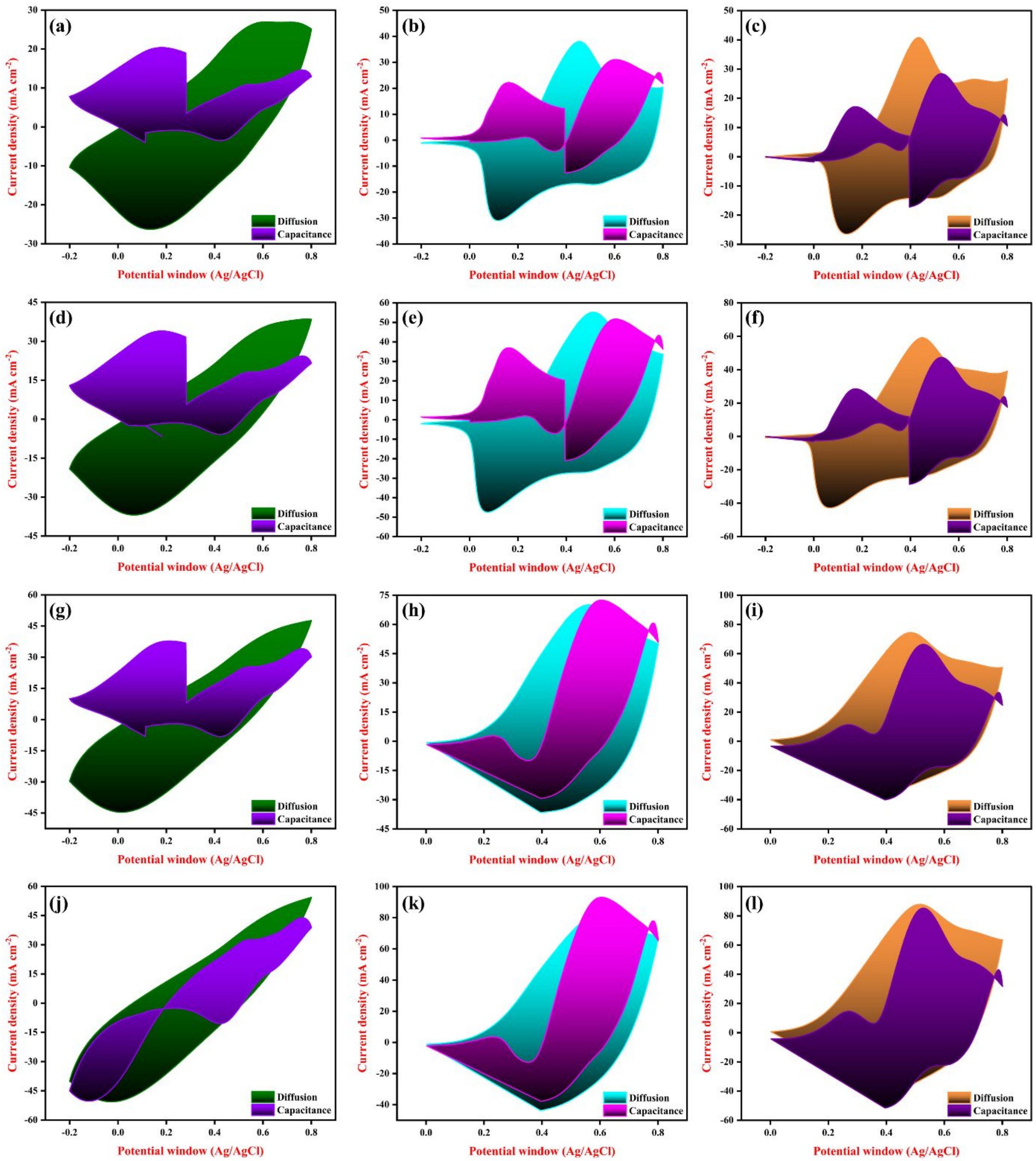

Figure S6. Dunn method: calculated from 30 mV/s, 50 mV/s, 70 mV/s, 90 mV/s, 100 mV/s (a, d, g, j) PANI, (b, e, h, k) PANI-Fe, and (c, f, i, l) PANI-Fe-CQD, respectively.

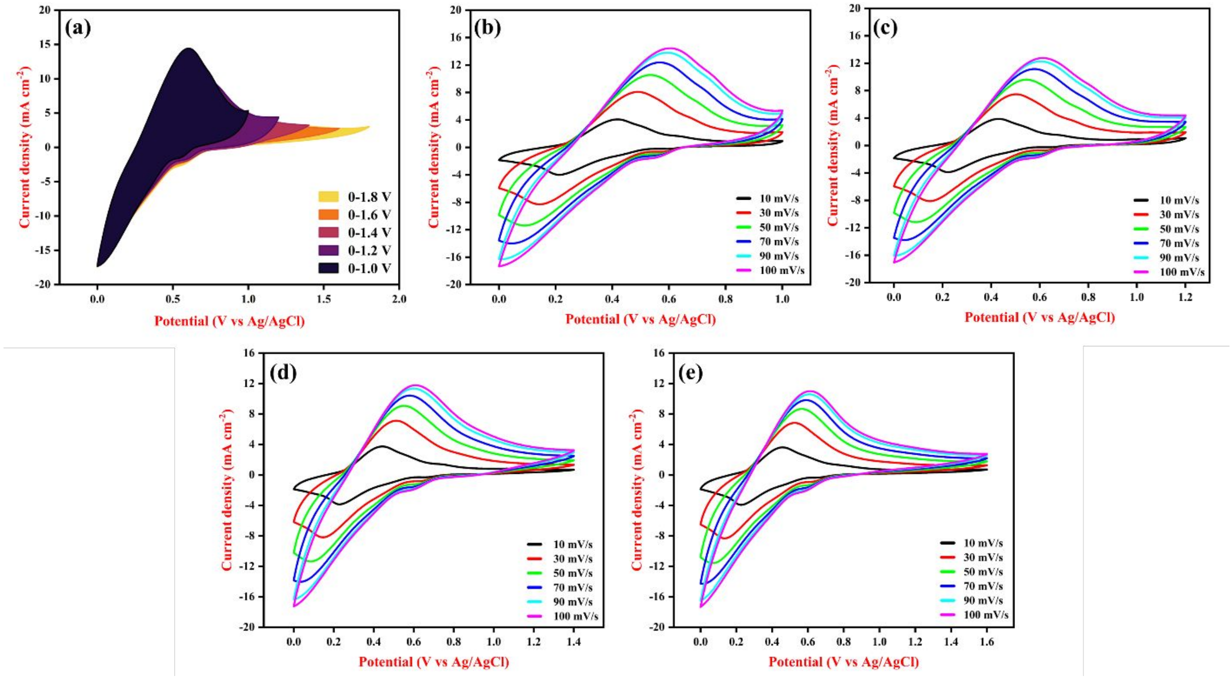

Figure S7. (a) CV different potential window analysis at 100 mV/s, and CV different scan rate studies on potential windows (b) 0- 0.1 V, (c) 0-1.2 V, (d) 0-1.4 V. and (e) 0-1.6 V.

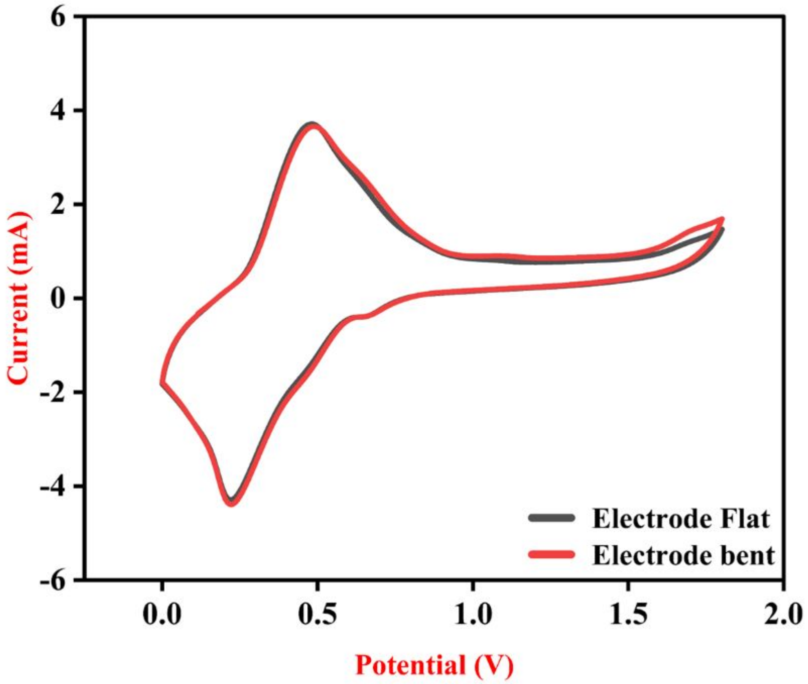

Figure S8. The CV studies of the fabricated AC//PVA-H_2SO_4//PANI-Fe-CQD at 10 mV/s.

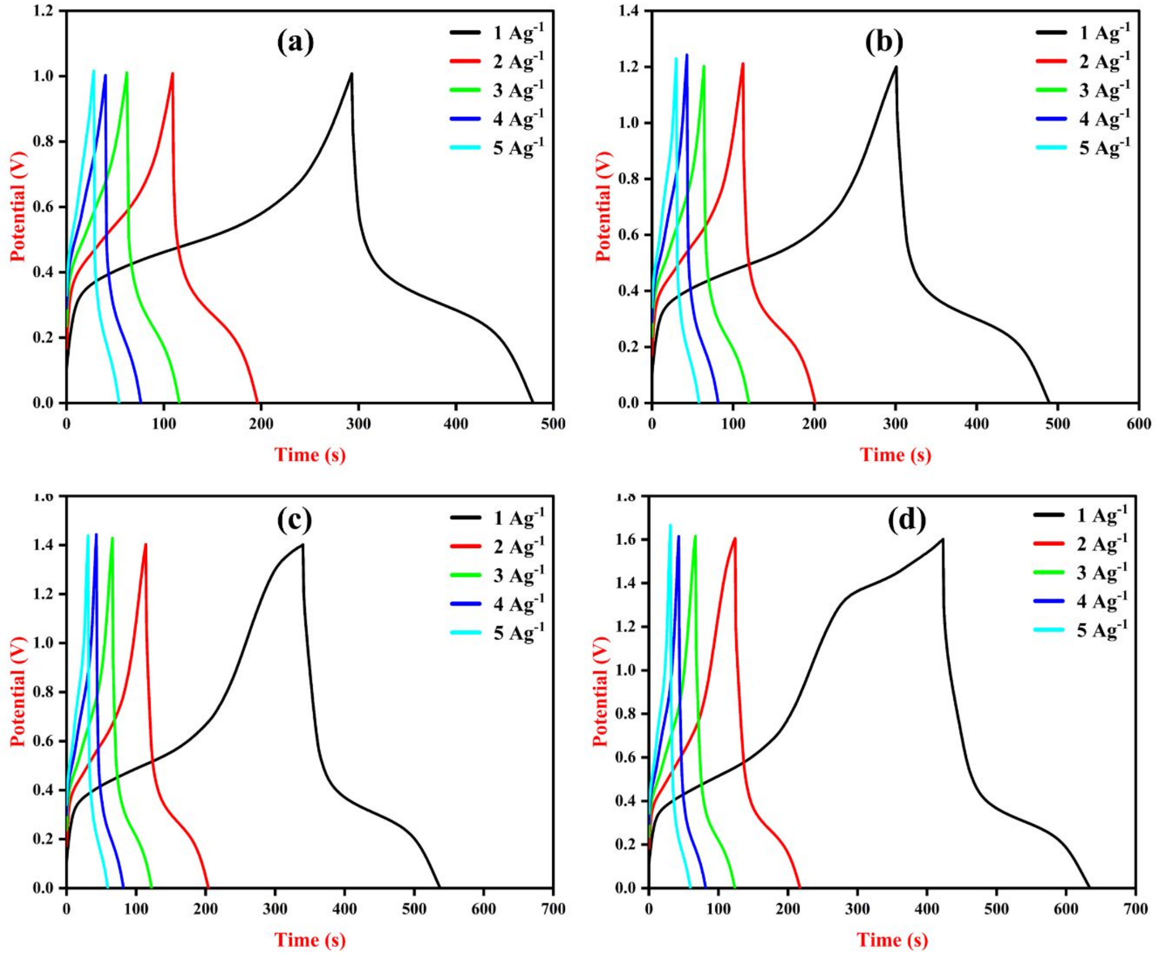

Figure S9. GCD analysis on different current densities over different potential windows (a) 0-1.0 V, (b) 0-1.2 V, (c) 0-1.4 V, and (d) 0-1.6 V.
